# Analysis of Ecuador's SCOPUS scientific production during the 2001–2020 period by means of standardized citation indicators

**DOI:** 10.1016/j.heliyon.2022.e09329

**Published:** 2022-04-25

**Authors:** V. Rodríguez, M. Flores-Sanchez, C.H. Zambrano, L. Rincón, J.L. Paz, F.J. Torres

**Affiliations:** a Grupo de Quimica Computacional y Teorica (QCT-USFQ), Departamento de Ingenieria Quimica, Universidad San Francisco de Quito (USFQ), Diego de Robles y Via Interoceanica, Quito, 17-1200-841, Ecuador; b Departamento de Matemática, Universidad San Francisco de Quito (USFQ), Diego de Robles y Vía Interoceánica, Quito, 17-1200-841, Ecuador; c Departamento Académico de Química Inorgánica, Facultad de Química e Ingeniería Química, Universidad Nacional Mayor de San Marcos, Lima, Peru; d Grupo de Química Computacional y Teórica (QCT-UR), Facultad de Ciencias Naturales, Universidad del Rosario, Bogotá, Colombia

**Keywords:** Composite indicator, Ecuador, Science, Technology, and innovation, ICSR

## Abstract

An analysis of the scientific production of Ecuador is performed by means of the composite indicator computed for Ecuador-based authors as compared to their counterparts of other South American countries. The dataset employed was obtained from the Databricks platform of the ELSEVIER's International Center for Science Research, ICSR. Therefore, this analysis is limited to the metadata of the documents published in journals indexed in SCOPUS. Comparison of the results obtained for two decades: 2001–2010 and 2011–2020 showed that the number of Ecuador-based researchers has significantly increased in different areas of knowledge. Moreover, comparison between the total number of authors that worked in Ecuador at any given year of the 2011–2020 period and the number of authors that are still working in this country up to the date of the data extraction (i.e., June 2021) showed an average of ∼68% of permanency. Analysis of the percentage distribution in terms of range quarters of the composite indicator (i.e., Q4: 0–1.5, Q3: 1.5–3.0, Q2: 3.0–4.5, and Q1: 4.5–6.0) showed that nearly the totality of the Ecuador-based researchers has composite indicators that lay in the Q4 and Q3 ranges for all the scientific fields considered. The latter was observed to be an effect of the scientific impact of South American countries, with larger investments in science and technology in comparison to Ecuador (i.e., Argentina, Brazil, and Chile). Exclusion of this group of countries in the calculation of the composite indicator of Ecuador-based authors resulted in a noticeable increment of scientists with composite indicators within Q2. Finally, our results suggest, in agreement with previous studies, a correlation between the sustained growth of scientific productivity in the decade 2011–2020 with the scientific programs and policies created by the state, where the initiative of scientific culture is shown as a strategy for growth and development.

## 1 Introduction

As indicated in the 2021 UNESCO Science Report, the countries belonging to South America are characterized by historic low investments in science, technology, and innovation (STI) [1]. Accordingly, this region registers a modest scientific production in comparison to its northern counterparts. As inferred from the SCOPUS database (see Table S1 in Supporting Information) and shown by Noorden [2], South American share of the world's scientific publications between 2001 and 2010 amounts to ∼2.6%, and although the scientific outcome of the region has increased to a share of 3.8% during the subsequent 2011–2020 period (Table S1), it is still considered to underperform relative to its ∼6% share of the global population [2, 3, 4]. Apart from the limited investment in STI, other reasons have been suggested for the low scientific production of South American countries. The significantly small university-educated graduates to the overall population ratio, the historic low number of research projects proposed and executed, the lack of national research facilities, and the limited supply of post-graduate students are some of them [5].

**Figure 1:**
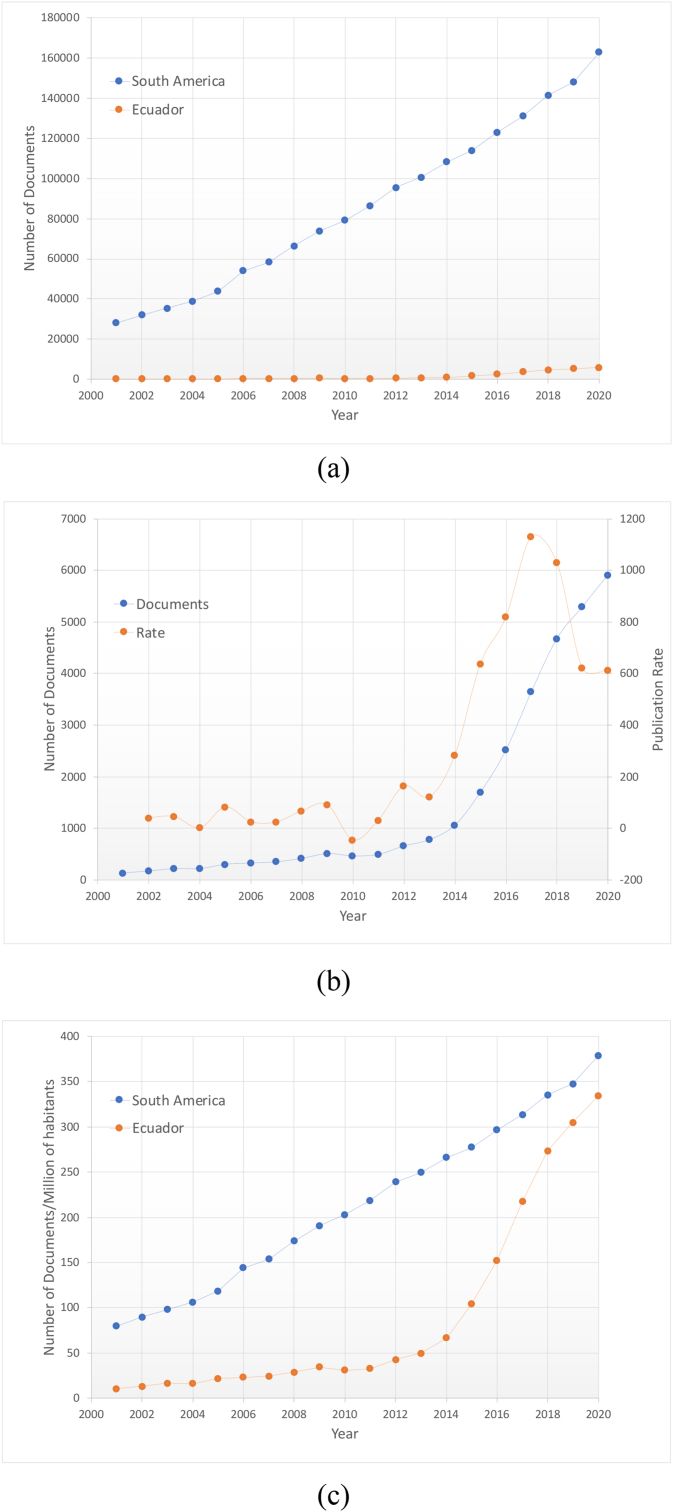
(a) Comparison of the scientific documents published in SCOPUS journals by Ecuadorian researchers and their South American counterparts. (b) Number of documents and publication rate of Ecuadorian researchers. (c) Same data as in the upper panel expressed in number of documents per million of habitants. All the data corresponds to the 2001-2020 period.

Ecuador, a South American country with a middle-income economy, is not an exception within the region, and it reports a scarce scientific production. As depicted in Figure 1a, the scientific output of this country in SCOPUS journals represents only a small fraction of the already low South American outcome. As also observed in Table S1, the documents published by Ecuador-based authors amounts only to about ∼2% of the region's total number of documents produced in the first two decades of the 2000s, which would be related to a modest development in STI [6]. Similar conclusions emerge when the number scientific documents relative to the total population is analyzed. As summarized in Table S2 (see Supporting Information), the scientific production of Ecuador is between the 10 and 67 documents per million inhabitants during the 2001–2014 period, representing respectively ∼1/8 and ∼1/4 of the value estimated for the whole South American region.

In order to improve the research conditions in Ecuador, a number of strategies and public policies aimed to stimulate the scientific development were implemented in Ecuador during the first decade of the present century, as part of an ambitious governmental plan, titled “*Plan Nacional del Buen Vivir”* (national plan of good living) or “*Sumak Kawsay”*, meant to improve the quality of life of Ecuadorians [7]. As an example of the latter, the Secretariate of Higher Education, Science, Technology and Innovation (Secretaría de Educación Superior, Ciencia, Tecnología e Innovación, SENESCYT) established the Prometheus Program, whose execution sought to incorporate internationally recognized scientists of different scientific fields within the Ecuadorian research apparatus (i.e., national research institutes, colleagues, and universities) by means of significant economic incentives, such as high salaries and a number of subsidies intended to increase the scientific production [8]. Another important example was the implementation of a public scholarship program designed to financially support the superior studies of Ecuadorians in recognized universities and research institutes around the world. As reported by Chávez and Gaybor in a recent study on the role of scientific networking in the publication trends of Ecuador [9], about ∼8000 Ecuadorians have directly benefited from the public scholarship program by 2019. Of this total, it is estimated that approximately ∼88% of the scholarships were used by the beneficiaries to pursuit either a master or a PhD title. These actions appear to have had a positive impact in the scientific production of Ecuador as inferred by observing the increase of the number of publications of Ecuador-based authors in journals indexed in SCOPUS as well as the publication rate during the 2011–2020 period in comparison to the previous decade (see Figure 1b). The same trend is observed regarding the data on the scientific articles relative to population reported in Table S2, where it is shown that this indicator exceeded the 100 documents per million inhabitants in 2015, and it was about to reach the South American average in 2020. From Table S2 and Figure 1c, it is also observed that Ecuador's per capita publication has grown from 15% of the South American average in the 2001–2010 decade to an impressive 89% in the 2011–2020 period, a fact that suggests a remarkable evolution of the scientific production of the country. It is important to note that similar observations about the growth of the scientific production of Ecuador were reported and discussed in previous studies, where the scientific articles published in different periods of time (i.e., 1920–2020 in Ref. [10] and 2006–2015 in Ref. [11]) were analyzed by means of various bibliometric tools. Here, a further analysis of the scientific outcome of Ecuador is proposed as a complement to previous studies [9, 10, 11]. In this context, the present work has two main objectives: (i) to compute the composite indicator of Ecuador-based authors that have published scientific manuscripts in journals indexed in the SCOPUS database and (ii) to obtain a diagnosis of the scientific production of Ecuadorian researchers as contrasted to their South American counterparts by means of the analysis of this standardized citation indicator. Although an assessment of the full STI development of a country is a difficult task that deserves the inclusion of other descriptors apart from the scientific production and involves several geographical, geopolitical, and social-economic factors (among others), this study is expected to provide further insights on the current scientific situation of Ecuador. At the same time, this study does not seek to probe the effect of each public policy adopted in Ecuador to stimulate the development of STI, but to provide a general view of the impact of the whole set of the strategies implemented during the first decade of the 21^st^ century. For this purpose, two periods of time are considered; namely: 2001–2010 and 2011–2020; hereinafter referred to as decade-A and decade-B, respectively. Moreover, it must be pointed out that the composite indicator is evaluated in the context of the South America region to avoid an unfair comparison with countries that have large investments in STI (i.e., north hemisphere nations) [12]. The latter means that only the SCOPUS production of authors that belong to Argentina, Bolivia, Brazil, Chile, Colombia, Ecuador, Paraguay, Perú, Uruguay, and Venezuela is considered in the present study. Finally, the Ecuadorian as well as the South American researchers are classified according the 26 different scientific fields defined by SCOPUS (see Table 1), spanning the knowledge generation of four main areas: (i) Health Sciences, (ii) Life Sciences, (iii) Physical Sciences, and (iv) Social Sciences. To the best of the authors' knowledge, the present study contrasts for the first time, and in a quantitative way, the scientific outcome of Ecuadorian researchers with their South American counterparts. It must be also mentioned that, through this indicator, we intend to somehow obtain a measure of the scientific production and quality of research, together with the leadership represented by the factors associated with the position of the authors within the articles (see Methodology Section). Considering both major factors, it would be possible to estimate the growth of scientific production, as well as to motivate collaborations that, through the programs applied in the latter part of the first decade, are still being maintained in Ecuador.

**Table 1:** Number of the Ecuador-based authors in the A1 as well as A2 datasets for the different scientific fields F and decades considered in the present study as extracted from the ELSEVIER laboratory of the International Center for the Study of Research (ICSR). The percentage of permanency and the increment of authors are also reported for each scientific field.

F	Decade-A: 2001–2010			Decade-B: 2011–2020			Increment	
	A1	A2∗	%Perm.	A1	A2∗	%Perm.	A1	A2
AGRI: Agricultural and Biological Sciences	301	160	53.2	894	516	57.7	2.0	2.2
ARTS: Arts and Humanities	4	1	25.0	33	17	51.5	7.3	16.0
BIOC: Biochemistry, Genetics, and Molecular Biology	44	26	59.1	165	94	57.0	2.8	2.6
BUSI: Business, Management, and Accounting	1	1	100	130	96	73.8	129.0	95.0
CENG: Chemical Engineering	1	0	0.0	25	14	56.0	24.0	-
CHEM: Chemistry	8	7	87.5	91	52	57.1	10.4	6.4
COMP: Computer Science	49	47	95.9	1155	1082	93.7	22.6	22.0
DECI: Decision Sciences	0	0	-	8	6	75.0	-	-
DENT: Dentistry	0	0	-	9	4	44.4	-	-
EART: Earth and Planetary Sciences	92	43	46.7	200	116	58.0	1.2	1.7
ECON: Economics, Econometrics, and Finance	5	2	40.0	26	14	53.8	4.2	6.0
ENER: Energy	5	3	60.0	98	67	68.4	18.6	21.3
ENGI: Engineering	33	21	63.6	391	277	70.8	10.8	12.2
ENVI: Environmental Sciences	111	55	49.5	307	167	54.4	1.8	2.0
HEAL: Health Professions	0	0	-	6	6	100.0	-	-
IMMU: Immunology and Microbiology	22	16	72.7	65	36	55.4	2.0	1.3
MATE: Materials Science	9	6	66.7	78	49	62.8	7.7	7.2
MATH: Mathematics	12	7	58.3	107	85	79.4	7.9	11.1
MEDI: Medicine	375	220	58.7	982	606	61.7	1.6	1.8
NEUR: Neuroscience	17	17	100	35	28	80.0	1.1	0.7
NURS: Nursing	0	0	-	10	7	70.0	-	-
PHAR: Pharmacology, Toxicology, and Pharmaceutics	9	9	100	109	74	67.9	11.1	7.2
PHYS: Physics and Astronomy	44	15	34.1	220	86	39.1	4.0	4.7
PSYC: Psychology	2	1	50.0	22	15	68.2	10.0	14.0
SOCI: Social Sciences	29	9	31.0	338	223	66.0	10.7	23.8
VETE: Veterinary	5	3	60.0	64	45	70.3	11.8	14

∗ Ecuador-based authors currently affiliated to an Ecuadorian institute when the present study was conducted (data extraction date: June 2021).

## 2 Methodology

As mentioned above, we have used the composite indicator as a metric tool to establish not only the quantitative aspects of scientific production, but also to link it with aspects of scientific impact and influence (as the composite indicator provides a measure of the citations received by the authors, see below) and to establish other byproducts as the parameters relative to mobility and permanence of scientists in the studied periods. As reported in Refs. [13, 14], the composite indicator for the *i-th* scientist (*c*_*i*_) in a defined group of authors is an index that takes values within the 0–6 range, and it is evaluated by considering six metrics, namely: the Hirsch h-index (*h*), the co-authorship-adjusted Schreiber hm-index (*hm*), the total number of citations (*NC*), the citations to papers as single author (*NCS*), the citations to papers as single or first author (*NCSF*), and the citations to papers as single, first or last author (*NCSFL*); which are combined through the following expression:

(1) $$c_{i}=\frac{\text{log}\left(h_{i}+1\right)}{\text{log}\left(h_{max}+1\right)}+\frac{\text{log}\left(hm_{i}+1\right)}{\text{log}\left(hm_{max}+1\right)}+\frac{\text{log}\left(NC_{i}+1\right)}{\text{log}\left(NC_{max}+1\right)}+\frac{\text{log}\left(NCS_{i}+1\right)}{\text{log}\left(NCS_{max}+1\right)}+\frac{\text{log}\left(NCSF_{i}+1\right)}{\text{log}\left(NCSF_{max}+1\right)}+\frac{\text{log}\left(NCSFL_{i}+1\right)}{\text{log}\left(NCSFL_{max}+1\right)}$$

It is important to point out that the maxima employed in the latter expression must be defined from a set of authors sharing a common characteristic. A usual practice is to consider authors belonging to the same scientific field where the publication rate as well as citation density are about the same [15]. Moreover, another condition could be imposed by considering authors belonging to the same country or geographical region, being therefore characterized by a similar social-economics situation [1, 16, 17]. In the present case, only the countries belonging to the South America region are considered.

**Figure 2:**
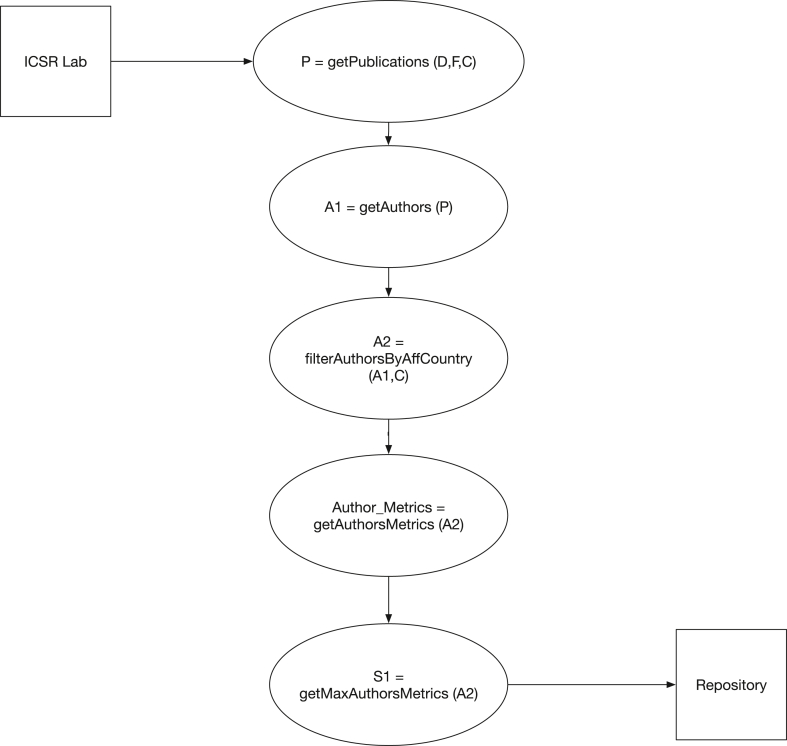
Schematic representation of the methodology applied in the present study to compute the composite indicator of Ecuador-based authors.

The data for the present study were obtained on June 2021 from the SCOPUS database by means of Python/PySpark scripts that make use of the Databricks platform as implemented in the ELSEVIER International Laboratory for the Study of Research (ICSR). As depicted in Figure 2, the developed scripts employ the following logic:

1. For a given decade (D), scientific field (F), and country (C), a complete list of publications (P) was obtained. It is important to indicate that discrimination between the type of documents (i.e., full paper, conference paper, review, communication, … etc) was not considered in the present study.
2. The whole set of authors of each publication in P was explored, and a preliminary list (A1) of the authors that belonged to the country C at any year of the decade D was produced. Furthermore, an author was included in the field F if his or her publication frequency was larger or equal to 3. Smaller frequency values were not employed since they resulted in many authors associated with more than one field.
3. The set A1 was subsequently refined by excluding all the authors whose current affiliation country is different to C to produce the final set A2.
4. For each author of set A2, the six metrics needed to compute the composite indicator according to Eq. (1) are either calculated or determined. The *h* and *hm* indexes were evaluated according to their definition as reported in Refs. [18, 19] only for the years comprised in decade D; whereas, the *NC*, *NCS*, *NCSF*, and *NCSFL* metrics were obtained including self-citations. In these regards, it is important to mention that the present study does not intend to evaluate the effect of the self-citation practice on the Ecuadorian scientific metrics.
5. As a final step, the maximum values of each metric were identified for each scientific field F and country C, and the composite indicator (*c*_*i*_) of the *i-th* author of set A2 was evaluated through Eq. (1). The maximum values of each metric for every field F and country C are summarized in Table S3 of the Supporting Information as Log(Max+1) as defined in Refs. [13, 14].

By following steps 1 to 5, the whole set of data employed in the present study was obtained where D = decade-A and decade-B, F = AGRI, ARTS, BIOC, BUSI, CENG, CHEM, COMP, DECI, DENT, EART, ECON, ENER, ENGI, ENVI, HEAL, IMMU, MATE, MATH, MEDI, NEUR, NURS, PHAR, PHYS, PSYC, SOCI, VETE, and C = Argentina, Bolivia, Brazil, Chile, Colombia, Ecuador, Paraguay, Perú, Uruguay, and Venezuela.

Before closing the present section, some comments regarding the adopted methodology must be pointed out:

1. By comparing the data obtained for decade-A and decade-B valuable insights on the increase of Ecuador-based authors, with production in each scientific field considered in the present study, are inferred by means of the following expression:

(2) $$Increment=\frac{A_{decadeB}-A_{decadeA}}{A_{decadeA}}$$

where, *A* refers to the number of authors in either A1 or A2 datasets.

2. By comparing the author sets A1 and A2 for each field, the number of scientists that have contributed to the development of science and are still working in Ecuador can be obtained. The latter appraisal is important to avoid the inclusion of researchers that worked in South American countries for short periods of time within any of the considered decades; for instance, scientists that have visited Ecuador on the frame of a sabbatical leave or some other scheme of research visit. The permanency of Ecuador-based authors in each field F was evaluated as follows:

(3) $$\%Permanency=\frac{A2}{A1}\times 100$$

3. Access to the data on the maximum values of the six metrics included in Eq. (1) for every scientific field F and country C allows the exclusion of South American nations, with much larger historic investments in STI in comparison to Ecuador (i.e., Argentina, Brazil, and Chile), in the evaluation of the composite indicator of Ecuador-based authors. This is expected to avoid unfair comparisons between Ecuador and countries that possess a more developed scientific apparatus [12].

## 3 Results

Table 1 summarizes the number of authors in sets A1 and A2 (see Methodology Section) for the different scientific fields and decades considered in the present study. From these data, it is observed that most of the scientific fields have a modest representation. By considering an average population of 13952723 registered for Ecuador in decade-A, the number of researchers per million inhabitants is inferior to 27 in all the fields, being negligible if it is compared to other South American countries such as Argentina and Brazil, where the number of scientists has been estimated to be approximately of 1207 and 888 researchers per million inhabitants, respectively, as indicated in the UNESCO's Science Report of 2021 for the same period [1]. ARTS, BUSI, CENG, CHEM, DECI, DENT, ECON, ENER, HEAL, MATE, MATH, NURS, PHAR, PSYC, VETE are the fields with the lowest absolute number of researchers, with a count of less than 10 authors. In contrast, MEDI and AGRI are the fields with the largest number of Ecuadorian scientists in decade-A, accounting for 375 and 301 researchers as reported for set A1, respectively. Likewise, the EART and ENVI fields show a significant count of authors, being close to 100 researchers; whereas PHYS is the only fundamental science with certain representation, having 44 authors within set A1.

Data reported for decade-B in Table 1 shows an important increase of the number of Ecuadorian authors in all the scientific fields. The increment in each scientific field computed through Eq. (2) for the A1 and A2 datasets are reported in the last columns of Table 1. These results indicate an impressive increment about ∼100 for BUSI, being the subject area with the largest increase of Ecuadorian authors. Other fields with significant growth are CENG, COMP, ENER with values close to ∼20 in terms of the A1 dataset, followed by CHEM, ENGI, PHAR, PSYC, SOCI, and VETE for which increments close to ∼10 is computed. Interestingly, the fields AGRI and MEDI, that report the highest Ecuadorian representation during decade-A, are among the fields with the lowest increase, being close to 2. It must be pointed out that the latter observations agree well with the growth of the SCOPUS production registered for Ecuador during the 2001–2020 period (see Figure 1b). However, more adequate insights on the increase of scientific documents produced by Ecuador-base scientists can be attained by estimating the documents to author ratio for each decade. According to the SCOPUS database, a total of 3106 and 26600 documents were produced in Ecuador during de decade-A and decade-B, respectively (Figure 1). The latter values result in averages of 2.6 and 4.8 documents/author for decade-A and decade-B, respectively, when the total of authors in the A1 dataset is considered. On the other hand, if the total of authors in the A2 dataset is considered instead, the ratios change to 4.6 and 7.0 documents/author for decade-A and decade-B, respectively. These results suggest that the difference between the sets A1 and A2 are relevant for the analysis of the present work; therefore, this will be discussed in more detail in the following paragraph.

As mentioned before, the set A1 accounts for the authors affiliated to Ecuador at any year or period within decade-A or decade-B; whereas A2 dataset considers the authors of A1 whose current affiliation is an Ecuadorian institution as obtained on June 2021 when the data extraction was performed from the ICSR. As commented before, the latter partition allows us to discriminate authors that temporarily worked in Ecuador (e.g., visiting scholars, foreign collaborators participating in short research stages, professors on sabbatical leaves, etc.) and authors that can be considered as long-lasting incorporations to the research apparatus of this country. These results on the permanency obtained through Eq. (3) are also reported in Table 1, where it can be observed that the percentage of permanency varies significantly along all fields if decade-A is considered; whereas, for decade-B, at least half of the authors that worked in Ecuador are still affiliated to an Ecuadorian institution nowadays for all the fields. In these regards, the HEAL field exhibits a percentage of permanency of 100% for decade-B; however, it must be considered that only 6 Ecuadorian authors have been classified inside this subject area. On the other hand, COMP is the area with the largest count of authors as well as percentage of permanency, which allows this field to be suggested as one of the most developed ones in Ecuador during the 2001–2020 period as also concluded by Herrera-Franco *et al.* [10]. Other fields presenting important percentages of permanency are: NEUR, MATH, DECI, BUSI, VETE, and NURS as obtained for decade-B. Finally, the percentage of permanency is about ∼68% on average for decade-B.

**Figure 3:**
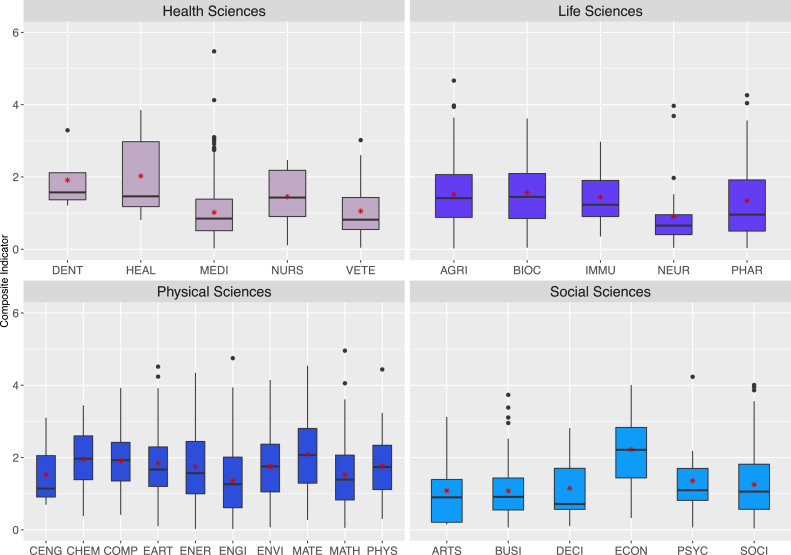
Statistical distribution around the median of the composite indicator computed for the Ecuador-based authors in the A2 dataset of decade-B. The scientific fields are grouped in four main areas of knowledge; namely: Health Sciences, Life Sciences, Physical Sciences, and Social Sciences.

Following the main objective of the present study, the composite indicator of every author belonging to A2 set was computed according to Eq. (1) as described in the Methodology Section. It is important to indicate that the discussion of this section focuses on data reported solely for decade-B because of the negligible Ecuadorian representation in some fields observed for decade-A. The distribution of the resulting values is depicted in Figure 3 for the different fields grouped in four main areas (as defined by SCOPUS); namely: Health Sciences, Life Sciences, Physical Sciences, and Social Sciences. As shown in Figure 3, the distributions are homogeneous in most of the cases, being exceptions of the latter HEAL, VETE, IMMU, PHAR, CENG, ARTS, DECI, and PSYC for which distributions skewed to higher values of the composite indicator are observed. From Figure 3, it is also observed that the field with the highest median ∼2.15 is ECON, which suggests that the scientific outcome of the Ecuadorian authors belonging to this area has important academic and scientific influence. Furthermore, the average of the composite indicator for authors in ECON coincides with the median value, determining an almost normal distribution for this specific case. In contrast, NEUR is the field with the lowest median value ∼0.65; nevertheless, the two highest outliers of this field are located at significant high values, close to 4.0.

In Health Sciences, MEDI is the scientific field with the largest number of Ecuadorian authors; accordingly, significant dispersion of data is observed in this case, and several outliers are reported at large values of the composite indicator. As a matter of fact, the Ecuadorian scientist with the highest value of the composite indicator (i.e., close to ∼5.5) belongs to the MEDI field, and this was also reported in a recent publication by Ioannidis and co-workers where a list of the 100000 top scientists worldwide was presented [14]. A similar behavior is observed for the AGRI field within the Life Sciences area. The latter case is characterized by a relatively small median value about ∼1.3 and, at the same time, several outliers located in the 4–4.7 range. Another field with some large outlier values of the composite indicator inside Life Sciences is PHAR, where two Ecuador-base authors exhibit values larger than 4. Concerning the Physical Sciences area, Figure 3 shows that the field with the largest median value is MATE followed by CHEM and COMP; however, EART, ENGI, PHYS, and MATH are the fields with Ecuador-based authors with the largest values of the composite indicator. Finally, the fields within the Social Sciences are characterized by relatively small median values of the composite indicator (an exception being the ECON field as commented before), suggesting that these fields are those with the Ecuador-based authors with the least scientific influence.

Since the definition of the composite indicator allows authors, that belong to different fields, to be directly compared, the data employed to produce Figure 3 can be distributed in range quarters; namely, Q4: [0.0–1.5[, Q3: [1.5–3.0[, Q2: [3.0–4.5[, and Q1: [4.5–6.0]. The count of authors as well as percentage distribution in terms of the latter ranges is summarized in Table 2, where it can be observed that the Ecuador-based authors have composite indicator values lying mainly in the Q4 and Q3 ranges for most of the fields. In fact, the Q4 percentage distribution is equal or greater than 75.0% of the total authors of the A2 dataset in the ARTS, BUSI, DENT, MEDI, NEUR, and VETE fields (bold characters in Table 2). On the other hand, the Q3 percentile distribution is larger than its Q4 counterpart in the CHEM, COMP, ECON, ENVI, MATE, and PHYS fields (bold characters in Table 2). Moreover, the EART, ENER, ENVI, HEAL, MATE, and PSYC fields have significant Q2 percentage distributions above 5.0%. Finally, only the MEDI field has a modest Q1 percentage distribution of 0.2% as expected by observing the outliers reported in Figure 3, associated to this field. The accumulation of the composite indicator values in the Q4 and Q3 ranges can be considered an effect of the scientific impact of South American countries with larger investments in STI as compared to Ecuador. In these regards, the data reported in Table S3 (see Supporting Information) show that the maxima employed for the calculation of the composite indicator through Eq. (1) are mainly defined by scientists of Argentina, Brazil, and Chile. Exclusion of these countries in the calculation of the composite indicator of Ecuador-based authors results in a re-distribution of the data. As reported in Table 2 (values in parentheses), a total of 164 scientists, belonging mainly to the AGRI, COMP, EART, and ENVI fields (data in bold characters), have now a composite indicator within the Q2 range. On the other hand, six different fields; namely: AGRI, EART, ENGI, MATE, MATH, and MEDI, report at least one author in the Q1 range. Thus, it can be suggested that some of the Ecuador-based authors belonging to the latter fields present significant leadership and impact.

**Table 2:** Count of authors and percentage distribution of the composite indicator computed for the Ecuador-based scientists of the A2 dataset and decade-B in terms of the range quarters: Q4, Q3, Q2, and Q1. Data in parenthesis are obtained by excluding Argentina, Brazil, and Chile in the calculation of the composite indicator of Ecuador-based authors. Bold characters represent cases where either the Q1 percentile distribution is higher than 75% or the Q2 percentile is larger than its Q1 counterpart.

Field	Count of Authors (#)				Percentage Distribution (%)			
	Q4: 0–1.5	Q3: 1.5–3.0	Q2: 3.0–4.5	Q1: 4.5–6.0	Q4: 0–1.5	Q3: 1.5–3.0	Q2: 3.0–4.5	Q1: 4.5–6.0
AGRI: Agricultural and Biological Sciences	289 (274)	211 (222)	**16 (19)**	**0 (1)**	56.0 (46.5)	40.9 (44.8)	3.1 (8.5)	0.0 (0.2)
ARTS: Arts and Humanities	13 (13)	4 (3)	0 (1)	0 (0)	**76.5 (76.5)**	23.5 (17.6)	0.0 (5.9)	0.0 (0.0)
BIOC: Biochemistry, Genetics, and Molecular Biology	51 (48)	41 (39)	1 (6)	0 (0)	54.8 (50.5)	44.1 (40.9)	1.1 (8.6)	0.0 (0.0)
BUSI: Business, Management, and Accounting	74 (74)	19 (19)	3 (3)	0 (0)	**77.1 (77.1)**	19.8 (19.8)	3.1 (3.1)	0.0 (0.0)
CENG: Chemical Engineering	9 (9)	5 (4)	0 (1)	0 (0)	64.3 (64.3)	35.7 (28.6)	0.0 (7.1)	0.0 (0.0)
CHEM: Chemistry	19 (16)	34 (35)	1 (3)	0 (0)	35.2 (27.8)	**63.0 (64.8)**	1.9 (7.4)	0.0 (0.0)
COMP: Computer Science	108 (87)	154 (164)	**7 (18)**	0 (0)	40.1 (32.3)	**57.2 (61.0)**	2.6 (6.7)	0.0 (0.0)
DECI: Decision Sciences	4 (4)	2 (2)	0 (0)	0 (0)	66.7 (66.7)	33.3 (33.3)	0.0 (0.0)	0.0 (0.0)
DENT: Dentistry	3 (2)	1 (1)	0 (1)	0 (0)	**75.0 (50.0)**	25.0 (25.0)	0.0 (25.0)	0.0 (0.0)
EART: Earth and Planetary Sciences	65 (51)	43 (46)	**8 (18)**	**0 (1)**	56.0 (44.0)	37.1 (39.7)	6.9 (15.5)	0.0 (0.0)
ECON: Economics, Econometrics, and Finance	6 (6)	8 (6)	0 (2)	0 (0)	42.9 (42.9)	**57.1 (42.9)**	0.0 (14.3)	0.0 (0.0)
ENER: Energy	33 (32)	26 (25)	8 (10)	0 (0)	49.3 (47.8)	38.8 (37.3)	11.9 (14.9)	0.0 (0.0)
ENGI: Engineering	186 (166)	84 (96)	7 (14)	**0 (1)**	67.1 (59.9)	30.3 (34.7)	2.5 (5.1)	0.0 (0.4)
ENVI: Environmental Sciences	74 (70)	84 (80)	**9 (17)**	0 (0)	44.3 (41.9)	**50.3 (47.9)**	5.4 (10.2)	0.0 (0.0)
HEAL: Health Professions	4 (3)	1 (1)	1 (2)	0 (0)	66.7 (50.0)	16.7 (16.7)	16.7 (33.3)	0.0 (0.0)
IMMU: Immunology and Microbiology	25 (21)	11 (15)	0 (0)	0 (0)	69.4 (58.3)	30.6 (41.7)	0.0 (0.0)	0.0 (0.0)
MATE: Materials Science	21 (13)	24 (25)	4 (10)	**0 (1)**	42.9 (26.5)	**49.0 (51.0)**	8.2 (20.4)	0.0 (2.0)
MATH: Mathematics	59 (47)	24 (32)	2 (5)	**0 (1)**	69.4 (55.3)	28.2 (37.6)	2.4 (5.9)	0.0 (1.2)
MEDI: Medicine	493 (476)	111 (125)	1 (4)	**1 (1)**	**81.4 (78.5)**	18.3 (20.6)	0.2 (0.7)	0.2 (0.2)
NEUR: Neuroscience	25 (24)	2 (2)	1 (2)	0 (0)	**89.3 (85.7)**	7.1 (7.1)	3.6 (7.1)	0.0 (0.0)
NURS: Nursing	4 (4)	3 (3)	0 (0)	0 (0)	57.1 (42.9)	42.9 (57.1)	0.0 (0.0)	0.0 (0.0)
PHAR: Pharmacology, Toxicology, and Pharmaceutics	52 (47)	20 (17)	2 (10)	0 (0)	70.3 (63.5)	27.0 (20.3)	2.7 (16.2)	0.0 (0.0)
PHYS: Physics and Astronomy	35 (33)	49 (47)	2 (6)	0 (0)	40.7 (38.4)	**57.0 (54.7)**	2.3 (7.0)	0.0 (0.0)
PSYC: Psychology	11 (10)	3 (4)	1 (1)	0 (0)	73.3 (66.7)	20.0 (26.7)	6.7 (6.7)	0.0 (0.0)
SOCI: Social Sciences	148 (145)	68 (68)	7 (10)	0 (0)	66.4 (64.1)	30.5 (30.0)	3.1 (5.8)	0.0 (0.0)
VETE: Veterinary	35 (34)	10 (10)	0 (1)	0 (0)	**77.8 (75.6)**	22.2 (22.2)	0.0 (2.2)	0.0 (0.0)
AVERAGE	71.0 (65.7)	40.1 (42.0)	3.1 (6.3)	0.0 (0.2)				

In order to put the previous numbers into the South American context, a similar distribution analysis was performed for the other 9 countries of the region without excluding any country in the calculations. The count of authors as well as the corresponding percentage distribution for decade-B in terms of the same range quarters (i.e., Q4-Q1) for the different scientific fields and countries is summarized in Table S4 (see Supporting Information). The reported data shows that the South American countries with important investments in STI [16] are characterized by significant author representations in the Q2 and Q1 range quarters. For instance, Brazil reports an average of ∼377 and ∼32 authors/field in the Q2 and Q1 ranges, respectively; followed by Argentina and Chile, which accounts for ∼74 and ∼7 authors/field for the same range quarters. Another interesting case is Colombia, whose Q2 author count is about ∼35.4 authors/field, whereas the average in Q1 is about ∼5 authors/field. In contrast, the data reported in Table 2 indicates that Ecuador possesses an average close to ∼3 authors/field for Q2 range, while its representation in the Q1 ranges is practically negligible.

At this point, an important question arises: how do the composite indicator of Ecuador-based researchers and its quarter distribution modify if the A1 dataset is considered instead? To answer this question, the steps described in the Methodology Section were repeated to compute the composite indicator of the South America-based researchers without excluding any author from the dataset of each country C (i.e., step 3 is overlooked). The results obtained for the A1 dataset of Ecuador-based (and South America-based) researchers are summarized in Table S5 (see Supporting Information), where two main effects of considering this set are observed: (i) the number of researchers in every range quarter (i.e., Q1-Q4) of each field increases and (ii) the percentage distribution in terms of the range quarters slightly changes. As reported in Table S5, the average population for Q4, Q3, Q2, and Q1 are 94, 76, 17, and 1 authors/field, respectively, which represents increases of 23.6, 36.3, 13.8 and 1.3 authors/field with respect to the values reported in Table 2 for the A2 dataset. The fields characterized by the greatest increments are AGRI, MEDI, COMP, ENVI, PHYS, ENGI, and SOCI. On the other hand, the DECI, DENT, HEAL, NEUR, NURS, and PSYC fields report negligible changes. Regarding the percentage distribution, the data of Table S5 indicates that the composite indicator of Ecuador-based scientists concentrates within the Q4 and Q3 ranges, an observation that also emerged from the analysis of the results obtained for the A2 dataset (see above). Notwithstanding, it is important to note that some fields report authors with composite indicators within Q2 or Q1 ranges. Examples of the latter are the CHEM, EART, ECON, ENVI, HEAL, and PSYC fields, where the sum of the Q2 and Q1 percentage distributions is above 15%. Although this represents a remarkable difference with respect to the results obtained for the A2 dataset, the general trends of the data reported for the Ecuador-based researchers as compared to their South American counterparts (Table S5) coincides with the description proposed for data of Table 2 (see above). In this vein, one can suggests that the main conclusions of the present work are somehow independent of the author dataset (i.e., A1 or A2) employed for the analysis.

## 4 Discussion

As it can be seen, the use of the composite indicator as proposed in the present work confers us the possibility of conducting an analysis of the evolution of the scientific production of Ecuador and other related byproducts as linked to the strategies and public policies taken to improve the national research apparatus. In these regards, there are several factors to consider. For instance, as a result of the state policies aimed at promoting research in all areas of knowledge, many universities obtained important results in terms of an increase in the number of indexed documents, a fact that explains the increase of documents reported in Figure 1b. Likewise, the competition that was established between the universities with the largest number of Prometheus researchers (see Introduction Section) increased the speed at which these publications were produced (see Figure 1b) given that the requirements of the program demanded a sustained production of publications indexed in recognized impact databases such as SCOPUS in order to renew the researchers' affiliations for additional academic years. In this context, many of the institutions enforced rules on their researchers so that the production would not only increase in number, but also that the rate of publication would be greater. It is important to point out that, although it is true that the increase in scientific production is also a global phenomenon, mainly related to the economic growth, the globalization process, the growing internationalization of research, among others; in the case of Ecuador, it is possible to differentiate this growth from the global one by observing the rate with which the number of publications increased, even in those areas in which scientific production was more depressed such as the fundamental fields (i.e., mathematics, chemistry, and physics). The latter agrees well with the conclusions of Castillo and Powell reported in Ref. [11].

There is no doubt that policies or strategies promoted by the state, such as the distribution in all public universities of highly qualified researchers, with proven experience in research and high collaboration contacts, and the SENESCYT scholarship program [9] were decisive steps for the increase in the number and rate of publications [8]. Indeed, our emphasis on highlighting the difference in scientific production in the indicated decades had the fundamental objective of emphasizing that, in decade B, the country was opening to a scientific culture not only in terms of production, but also in terms of internationalization and scientific collaboration as also stated by Chávez and Gaybor [9]. The way research management was conducted during the decade-B was close to proposals like those of developed countries, regarding the speed of administrative processes, the flow of researchers, the organization of high-level scientific events, and the increase of new and better graduate programs nationwide [20]. Some of these policies are still being maintained with the culture fostered by these programs, added to the return of many scholarship holders trained abroad with outstanding scientific profiles.

It is important to note that in our analysis of scientific productivity, one of the important elements to highlight is the permanence of the researcher in the country. During the decade-B, the country underwent an important scientific transformation, not only with the incorporation of scientific policies aimed at improving scientific productivity as aforementioned, but also at fostering a culture of knowledge society, scientific development plans, construction of infrastructures dedicated to the clear orientation of research and an open system of transformation in the scientific field. However, it must be indicated that the continuous rotation of scientists entering and leaving the system let many projects unfinished, and without timely prepared replacements. In the same vein, one of the major deficiencies in the STI plans during that decade was the lack of synchronization between the state as a provider of resources and the public universities as acceptors of that qualified workforce. In many cases, there was a total disconnection between the insertion of qualified Prometheus researchers or beneficiaries of the SENESCYT scholarship program and the opportunities that the academic institution could offer, a fact that resulted in the disengagement of researchers from the universities and their subsequent leaving of the country as observed in our results associated to the permanency of Ecuador-based authors. Moreover, although the state had the economic resources to foster this scientific culture, many of the Prometheus program researchers were passing through the academic institutions as part of their sabbatical stays at their home institutions, that is why the term permanence is vital to understand much of what happened.

We are aware that in some cases we have excluded researchers who for some reason did not publish from an Ecuadorian institution at the specific time when the consultation was made. However, we believe that doing it the way it is presented in this article opens the possibility to explore many elements that were presented almost simultaneously related to the increase of scientific productivity. At present, we are considering a more appropriate way to calculate the % of permanency by considering, for instance, the frequencies of reported affiliation per publication over the entire decade to exclude those cases characterized by the lowest frequencies. Finally, it is important to point out that our study shows that the increase in the number of publications was closely related to the increase in the number of Ecuador-based scientists due to the implemented public policies and initiatives [8, 9]. However, it must be considered that, during decade-B, Ecuadorian universities received a very high contingent of researchers, most of whom had stable and prolific research groups in their countries of origin. This fact concentrated the Ecuador scientific productivity in a sector of mostly foreign researchers, and not in a more linear distribution with all the permanent researchers of the Ecuadorian institutions.

As a final point in the present discussion, the distribution of the composite indicator in terms of range quarters (i.e., Q4, Q3, Q2, and Q1) must be briefly commented. It is well-known that Brazil dominates the scientific production of South America due to the enormous investments that this country has historically devoted in STI [21]. Thus, this country can be considered as a point of reference to assess the influence and quality of the scientific production of Ecuador-based authors. By considering that Brazil has a significant number of researchers with composite indicator within the Q2 and Q1 quarters, being the average values about ∼377 and ∼30 authors/field; whereas Ecuador accounts for about ∼3 authors/field in the Q2 range and null representation in the Q1 quarter, it is clear from our results that the science, technology, and innovation in Ecuador are still in a very early stage of development as already noted by Castillo and Powell [22]. The latter appraisal means that, although the increase of the number of academic products as well as the Ecuador-based researchers is an indication of some improvement of the national research apparatus achieved in this country, the influence, and the associated quality [23] of the Ecuadorian science has still room for improvement. It must be also pointed out that, by observing the data reported for the other South American countries reported in Table S4 (see Supporting Information), Ecuador can be suggested to have similar research conditions as Venezuela, being placed or ranked just below Perú and above Bolivia. This relative placement within the South American context is proposed as a point of reference for future analysis on the development of science and technology of this country. Finally, from our results using the composite indicator as a measure of scientific production in Ecuador for the indicated decades of studies and in line with Chávez and Gaybor [9], the critical mass of researchers coming from the Prometheus program and those resulting from the SENESCYT scholarship program implemented by the national government, contributed substantially to the increase in scientific production. With both initiatives of scientific production and public policies aimed at basic or applied research, cooperation between groups of researchers both locally and internationally was also favored. Furthermore, the public policies created by the SENESCYT during the second decade of study clearly allowed an incentive towards the realization of scientific activity as an attraction for the mobility and stay of national and international researchers as part of collaboration agreements. At the same time, SENESCYT created a very large set of initiatives with the advice of foreign and national researchers who formulated many program proposals aimed not only at improving scientific productivity rates, but also at "sowing" spaces that would highlight the importance of scientific research as an engine of growth and generator of knowledge.

## 5 Conclusions

This work shows how both: (i) the increase in research scientists in different fields of study in Ecuador presumably promoted by public initiatives such as the Prometheus and SENESCYT scholarship programs and (ii), as reported by Chávez and Gaybor [9], the consequent growth of local as well as international research networks have had a direct beneficial impact in the scientific productivity of the country. Furthermore, the data showed that in fields where the growth in the number of investigators has been modest, the increase in academic production was also small. Comparison of the results obtained for the two decades: 2001–2010 and 2011–2020 showed that a notable increase in the number of researchers and production occurred in Business, Management and Accounting, Computer Science, Social Sciences, Energy, Arts and Humanities, Psychology, Engineering, and Mathematics.

The composite indicator data, classified in range quarters (i.e., Q4: 0–1.5, Q3: 1.5–3.0, Q2: 3.0–4.5, and Q1: 4.5–6.0), was found to be very useful to compare researchers from a same field with their counterparts in other South American countries. Ecuador-based authors displayed composite indicator values lying mostly in the Q4 and Q3 ranges for most fields, where the most important contributions came from Medicine, followed by Agricultural and Biological Sciences, Earth and Planetary Sciences, Engineering, and Mathematics. In general, this analysis revealed that South American countries, presumably with greater investments in STI, such as Brazil, Argentina, and Chile, are at the head of research production, while Ecuador, being behind Colombia, Uruguay and Perú, is at the tail end along with countries such as Venezuela, Bolivia and Paraguay.

Finally, the study carried out through this standardized citation indicator allows a greater knowledge of the areas that in Ecuador have had considerable progress, either by the incorporation of scientists related to the highlighted areas through their processes of development and growth, or through linkages as established by the undertaken actions such as the Prometheus and the SENESCYT scholarship programs. It also clearly evidenced those areas of knowledge and population zones that have been neglected during both decades. The study shows the need to strengthen with new scientific policies that allow sustaining the scientific production that has been taking place in the last years, but urgently the need to strengthen the fourth level studies in the country, thus avoiding the loss of human resources that do not return to the country after completing their studies. Finally, it is important that our analysis suggests that the increase in the number of publications is not only the result of a greater number of scientists, but also the improvement of the conditions to produce science from Ecuador.

## Declarations

### Author contribution statement

V. Rodríguez: Performed the experiments.

M. Flores-Sanchez: Performed the experiments; Wrote the paper.

C. H. Zambrano: Conceived and designed the experiments; Wrote the paper.

L. Rincó: Conceived and designed the experiments; Analyzed and interpreted the data.

J. L. Paz: Analyzed and interpreted the data.

F. J. Torres: Conceived and designed the experiments; Analyzed and interpreted the data; Wrote the paper.

### Funding statement

This work was supported by Alianza EFI-Colombia Científica (grant codes: 60185 and FP44842-220-2018).

### Data availability statement

Data included in article/supplementary material/referenced in article.

### Declaration of interests statement

The authors declare no conflict of interest.

### Additional information

No additional information is available for this paper.

## Appendix A Supplementary data

SI SI
