# Supplementary material for: Analysis of Ecuador's SCOPUS scientific production during the 2001–2020 period by means of standardized citation indicators
Source: Heliyon. 2022 Apr 25;8(4):e09329. doi: 10.1016/j.heliyon.2022.e09329 (PMC9062253; doi:10.1016/j.heliyon.2022.e09329)
Supplement: SI [file mmc1.docx]

**Table S1.** World, South American, and Ecuador scientific production published in journals indexed in the SCOPUS database during the 2001-2020 period. The South American region is considered to include the following countries: Argentina, Bolivia, Brazil, Chile, Colombia, Ecuador, Paraguay, Perú, Uruguay, and Venezuela.

|  | **Number of Documents** | | |  | |  |
| --- | --- | --- | --- | --- | --- | --- |
| **Year** | **World** | **South America*** | **Ecuador** | **South America Share of**  **World’s Production (%)** | **Ecuador Share of**  **South America Production (%)** | |
| 2001 | 1419272 | 27367 | 133 | 1.9 | 0.5 | |
| 2002 | 1488762 | 31197 | 173 | 2.1 | 0.5 | |
| 2003 | 1573401 | 34347 | 218 | 2.2 | 0.6 | |
| 2004 | 1688808 | 37698 | 219 | 2.2 | 0.6 | |
| 2005 | 1925467 | 42425 | 301 | 2.2 | 0.7 | |
| 2006 | 2025744 | 52287 | 326 | 2.6 | 0.6 | |
| 2007 | 2128827 | 56367 | 350 | 2.6 | 0.6 | |
| 2008 | 2229597 | 64155 | 417 | 2.9 | 0.6 | |
| 2009 | 2347682 | 71334 | 508 | 3.0 | 0.7 | |
| 2010 | 2471522 | 76557 | 463 | 3.1 | 0.6 | |
| **TOTAL** | **19299082** | **493734** | **3108** | **2.6** | **0.6** | |
| 2011 | 2634238 | 83152 | 494 | 3.2 | 0.6 | |
| 2012 | 2765655 | 91743 | 657 | 3.3 | 0.7 | |
| 2013 | 2890115 | 96262 | 778 | 3.3 | 0.8 | |
| 2014 | 2933034 | 103617 | 1061 | 3.5 | 1.0 | |
| 2015 | 2934070 | 108456 | 1697 | 3.7 | 1.5 | |
| 2016 | 3036169 | 116670 | 2515 | 3.8 | 2.0 | |
| 2017 | 3122607 | 124111 | 3646 | 4.0 | 2.8 | |
| 2018 | 3248663 | 133599 | 4676 | 4.1 | 3.3 | |
| 2019 | 3398505 | 139247 | 5296 | 4.1 | 3.6 | |
| 2020 | 3551785 | 152358 | 5909 | 4.3 | 3.6 | |
| **TOTAL** | **30514841** | **1149215** | **26729** | **3.8** | **2.3** | |

*The number of SCOPUS documents of South America for a given year (Y) were obtained by performing a advanced search with the following string: AFFILCOUNTRY(ARGENTINA) OR AFFILCOUNTRY(BOLIVIA) OR AFFILCOUNTRY(BRAZIL) OR AFFILCOUNTRY(CHILE) OR AFFILCOUNTRY(COLOMBIA) OR AFFILCOUNTRY(ECUADOR) OR AFFILCOUNTRY(PARAGUAY) OR AFFILCOUNTRY(PERU) OR AFFILCOUNTRY(URUGUAY) OR AFFILCOUNTRY(VENEZUELA) AND PUBYEAR IS Y.

**Table S2.** South America and Ecuador scientific production per million of habitants published in journals indexed in the SCOPUS database during the 2001-2020 period. The South American region is considered to include the following countries: Argentina, Bolivia, Brazil, Chile, Colombia, Ecuador, Paraguay, Perú, Uruguay, and Venezuela.

|  | **Documents/Million Inhabitants** | |  |
| --- | --- | --- | --- |
| **Year** | **South America*** | **Ecuador** | **Ecuador to South America**  **ratio** |
| 2001 | 80 | 10 | 0.12 |
| 2002 | 90 | 13 | 0.15 |
| 2003 | 98 | 16 | 0.17 |
| 2004 | 106 | 16 | 0.15 |
| 2005 | 119 | 22 | 0.18 |
| 2006 | 144 | 23 | 0.16 |
| 2007 | 154 | 24 | 0.16 |
| 2008 | 174 | 29 | 0.17 |
| 2009 | 191 | 34 | 0.18 |
| 2010 | 203 | 31 | 0.15 |
| 2011 | 219 | 32 | 0.15 |
| 2012 | 239 | 42 | 0.18 |
| 2013 | 249 | 50 | 0.20 |
| 2014 | 266 | 67 | 0.25 |
| 2015 | 277 | 105 | 0.38 |
| 2016 | 297 | 153 | 0.50 |
| 2017 | 313 | 217 | 0.70 |
| 2018 | 335 | 274 | 0.82 |
| 2019 | 348 | 305 | 0.89 |
| 2020 | 379 | 335 | 0.89 |

*The number of SCOPUS documents of South America for a given year (Y) were obtained by performing an advanced search with the following string: AFFILCOUNTRY(ARGENTINA) OR AFFILCOUNTRY(BOLIVIA) OR AFFILCOUNTRY(BRAZIL) OR AFFILCOUNTRY(CHILE) OR AFFILCOUNTRY(COLOMBIA) OR AFFILCOUNTRY(ECUADOR) OR AFFILCOUNTRY(PARAGUAY) OR AFFILCOUNTRY(PERU) OR AFFILCOUNTRY(URUGUAY) OR AFFILCOUNTRY(VENEZUELA) AND PUBYEAR IS Y. The population of each country for a given year (Y) was obtained from The World Bank database.

**Table S3.** Maximum value of the six metrics (NC, H, Hm, NCS, NCSF, and NCSFL) obtained for each country and field according to the SCOPUS production of the 2011-2020 period. Values are reported as Log (Max+1) as required for the calculation of the composite indicator through Eq. (1).

| **FIELD** | **METRIC** | **COUNTRY** | | | | | | | | | |
| --- | --- | --- | --- | --- | --- | --- | --- | --- | --- | --- | --- |
|  |  | **ARG** | **BOL^*^** | **BRA** | **COL** | **CHI** | **ECU** | **PAR^*^** | **PER** | **URU** | **VEN** |
| AGRI | NC | 4.0758752950 | 3.5440680440 | 3.8758711910 | 3.4673120630 | 3.7818271530 | 3.5435714240 | 2.7972675410 | 3.5280163410 | 3.7672300980 | 3.1513698500 |
|  | H | 1.5797835970 | 1.4623979980 | 1.6901960800 | 1.4313637640 | 1.5314789170 | 1.4623979980 | 1.1461280360 | 1.4471580310 | 1.6334684560 | 1.1760912590 |
|  | Hm | 1.1731052960 | 0.8309902300 | 1.2902806870 | 1.0096775380 | 1.1802578670 | 1.1357382560 | 0.7206760130 | 1.0096775380 | 1.3823535920 | 0.8737187760 |
|  | NCS | 2.6304278750 | 1.2304489210 | 3.4966529390 | 2.3502480180 | 3.5415792440 | 2.3242824550 | 1.3222192950 | 1.8750612630 | 1.9294189260 | 1.9395192530 |
|  | NCSF | 3.4625477290 | 2.4548448600 | 3.4966529390 | 3.0899051110 | 3.5613399410 | 2.9227254580 | 2.1931245980 | 2.8215135280 | 3.2540644530 | 2.5263392770 |
|  | NCSFL | 3.5476516580 | 2.5987905070 | 3.5544891600 | 3.0913151600 | 3.5963771440 | 3.1535099890 | 2.3820170430 | 2.9410142440 | 3.6566730460 | 2.5263392770 |
| ARTS | NC | 2.1818435880 | 1.3979400090 | 2.5820633630 | 2.7403626890 | 2.7299742860 | 1.5051499780 | 0.3010299960 | 2.6020599910 | 1.8920946030 | 2.0899051110 |
|  | H | 0.9542425090 | 0.4771212550 | 1.1461280360 | 0.8450980400 | 1.1139433520 | 0.6020599910 | 0.3010299960 | 0.9030899870 | 0.6020599910 | 0.8450980400 |
|  | Hm | 0.8450980400 | 0.6020599910 | 0.9664676570 | 0.8450980400 | 0.8702087610 | 0.6020599910 | 0.4771212550 | 0.6020599910 | 0.6989700040 | 0.8450980400 |
|  | NCS | 1.9294189260 | 1.0791812460 | 2.1492191130 | 1.9444826720 | 1.9190780920 | 1.2552725050 | 0.3010299960 | 1.2552725050 | 1.3802112420 | 1.8260748030 |
|  | NCSF | 2.0530784430 | 1.0791812460 | 2.2988530760 | 2.7403626890 | 2.5037906830 | 1.4149733480 | 0.3010299960 | 1.5051499780 | 1.6127838570 | 2.0043213740 |
|  | NCSFL | 2.1172712960 | 1.0791812460 | 2.4698220160 | 2.7403626890 | 2.5550944490 | 1.5051499780 | 0.3010299960 | 1.6334684560 | 1.8864907250 | 2.0899051110 |
| BIOC | NC | 3.9445320210 | 3.0211892990 | 4.3686215440 | 4.1085650240 | 4.2230544130 | 3.6155292240 | 2.3579348470 | 3.8246464150 | 3.7049222910 | 3.6593456360 |
|  | H | 1.6127838570 | 1.1461280360 | 1.6232492900 | 1.6627578320 | 1.7242758700 | 1.3010299960 | 0.9542425090 | 1.4471580310 | 1.5910646070 | 1.1760912590 |
|  | Hm | 1.1796745180 | 0.7829217250 | 1.2848069990 | 1.2330905790 | 1.3375198870 | 1.0582831870 | 0.6350722880 | 0.9847555540 | 1.2106767920 | 0.7357405720 |
|  | NCS | 2.5670263660 | 0.9542425090 | 2.3384564940 | 1.8512583490 | 3.2626883440 | 2.0644579890 | 0.9030899870 | 1.2304489210 | 2.9493900070 | 1.1139433520 |
|  | NCSF | 3.0034605320 | 1.7634279940 | 3.1942367490 | 2.7759743310 | 3.6303261550 | 2.5065050320 | 1.7923916890 | 2.5224442340 | 2.9503648540 | 2.2201080880 |
|  | NCSFL | 3.4599952560 | 2.2329961100 | 3.5590683340 | 3.5112147010 | 3.9303376360 | 2.7656685550 | 1.7923916890 | 2.8733206020 | 3.3434085940 | 2.4048337170 |

**Table S3.** Continue.

| **FIELD** | **METRIC** | **COUNTRY** | | | | | | | | | |
| --- | --- | --- | --- | --- | --- | --- | --- | --- | --- | --- | --- |
|  |  | **ARG** | **BOL^*^** | **BRA** | **COL** | **CHI** | **ECU** | **PAR^*^** | **PER** | **URU** | **VEN** |
| BUSI | NC | 2.8674674880 | 1.9395192530 | 3.3132342920 | 2.7781512500 | 2.9772662120 | 2.6875289610 | - | 3.3222192950 | 2.4082399650 | 1.8864907250 |
|  | H | 1.1461280360 | 0.6020599910 | 1.3979400090 | 1.1139433520 | 1.2787536010 | 0.8450980400 | - | 1.3979400090 | 1.0413926850 | 0.6989700040 |
|  | Hm | 1.0851716100 | 0.4648867980 | 1.2205439080 | 0.8972342420 | 1.1233070710 | 0.7659167940 | - | 1.2081725270 | 0.7201593030 | 0.6690067810 |
|  | NCS | 2.4031205210 | 0.3010299900 | 2.4232458740 | 1.6334684560 | 2.0530784430 | 1.6627578320 | - | 2.3344537510 | 1.2041199830 | 0.9542425090 |
|  | NCSF | 2.6364878960 | 0.6020599910 | 3.0269416280 | 2.6473829700 | 2.8494194140 | 2.6720978580 | - | 3.0791812460 | 1.9138138520 | 1.7993405490 |
|  | NCSFL | 2.6946051990 | 1.3979400090 | 3.2350231590 | 2.6937269490 | 2.9237619610 | 2.6730209070 | - | 3.2697463730 | 2.4082399650 | 1.7993405490 |
| CENG | NC | 3.0948203800 | 1.9493900070 | 3.8226910110 | 3.5425764760 | 3.4983105540 | 2.5010592620 | 2.9041743680 | 2.4440447960 | 2.4578818970 | 2.7109631190 |
|  | H | 1.3010299960 | 0.8450980400 | 1.5682017240 | 1.5185139400 | 1.3979400090 | 0.9030899870 | 1.2304489210 | 0.9030899870 | 1.0791812460 | 1.1461280360 |
|  | Hm | 1.0198083930 | 0.3010299900 | 1.2649114750 | 1.2399545690 | 1.0198083930 | 0.5554399490 | 1.0484017230 | 0.6130359870 | 0.6973123900 | 0.7788687370 |
|  | NCS | 1.0791812460 | 0.3010299900 | 1.6812412370 | 1.7160033440 | 1.3617278360 | 1.3979400090 | 2.0934216850 | 0.3010299900 | 0.3010299900 | 1.0791812460 |
|  | NCSF | 2.4712917110 | 1.6812412370 | 3.2284003590 | 3.1577588860 | 2.7604224830 | 2.2624510900 | 2.1731862680 | 2.0606978400 | 2.1038037210 | 1.8808135920 |
|  | NCSFL | 2.8943160630 | 1.6812412370 | 3.5514499980 | 3.3562171340 | 2.8457180180 | 2.3053513690 | 2.4232458740 | 2.1789769470 | 2.2944662260 | 2.5514499980 |
| CHEM | NC | 3.3975924340 | 1.9444826720 | 3.8379039450 | 3.5005109110 | 3.2977605110 | 3.0838608010 | 2.1205739310 | 2.7193312870 | 3.1958996520 | 3.3839947890 |
|  | H | 1.4149733480 | 0.7781512500 | 1.6020599910 | 1.4913616940 | 1.3617278360 | 1.3010299960 | 0.7781512500 | 1.1760912590 | 1.3617278360 | 1.3010299960 |
|  | Hm | 1.1887276540 | 0.4340363540 | 1.2703597840 | 1.2324515400 | 1.1985817190 | 0.9773424780 | 0.2124412810 | 0.9086339280 | 1.3117538610 | 0.8926245530 |
|  | NCS | 2.5403294750 | 0.3010299900 | 2.5599066250 | 1.5185139400 | 2.4297522800 | 0.3010299900 | 0.3010299900 | 0.3010299900 | 2.8115750060 | 1.1139433520 |
|  | NCSF | 2.9268567090 | 1.6627578320 | 3.0633333590 | 2.9484129660 | 2.8273692730 | 3.0386201620 | 0.3010299960 | 2.2648178230 | 3.0989896390 | 2.5037906830 |
|  | NCSFL | 3.2482185610 | 1.6627578320 | 3.6490426340 | 3.2172206560 | 3.0674428430 | 3.0390173220 | 0.3010299960 | 2.5132176000 | 3.1095785470 | 2.7176705030 |
| COMP | NC | 3.3706980930 | 2.2148438480 | 4.0884550450 | 3.9021661290 | 3.5048784590 | 3.2208922490 | 2.7923916890 | 2.7015679850 | 3.0305997220 | 3.1781132520 |
|  | H | 1.2787536010 | 0.9030899870 | 1.7403626890 | 1.4623979980 | 1.5051499780 | 1.3010299960 | 1.0791812460 | 1.1139433520 | 1.2552725050 | 1.2787536010 |
|  | Hm | 1.0075205600 | 0.6611814430 | 1.4787897350 | 1.1111904770 | 1.3239392750 | 0.9645738810 | 0.8543060420 | 0.9622114390 | 1.0016684800 | 1.0845762780 |
|  | NCS | 1.3979400090 | 0.6989700040 | 2.6683859170 | 1.7323937600 | 2.3242824550 | 1.1760912590 | 0.7781512500 | 2.0863598310 | 1.8573324960 | 1.6901960800 |
|  | NCSF | 3.1218879850 | 1.9637878270 | 2.9916690070 | 3.5827449660 | 2.9809119380 | 2.7209857440 | 2.2304489210 | 2.5289167000 | 2.4668676200 | 2.7671558660 |
|  | NCSFL | 3.1309766920 | 2.0253058650 | 3.7812525940 | 3.5873741720 | 3.3057811510 | 2.7466341990 | 2.6085260340 | 2.6512780140 | 2.7853298350 | 2.9420080530 |

**Table S3.** Continue.

| **FIELD** | **METRIC** | **COUNTRY** | | | | | | | | | |
| --- | --- | --- | --- | --- | --- | --- | --- | --- | --- | --- | --- |
|  |  | **ARG** | **BOL^*^** | **BRA** | **COL** | **CHI** | **ECU** | **PAR^*^** | **PER** | **URU** | **VEN** |
| DECI | NC | 1.9030899870 | - | 3.3074960380 | 3.1325798480 | 2.7795964910 | 1.7481880270 | - | 2.7067177820 | 0.8450980400 | 0.6989700040 |
|  | H | 0.7781512500 | - | 1.3424226810 | 1.2304489210 | 1.2041199830 | 0.6989700040 | - | 1.1139433520 | 0.3010299960 | 0.3010299960 |
|  | Hm | 0.3010299900 | - | 1.0803859470 | 0.9225524670 | 1.0142404390 | 0.4856538370 | - | 0.9098233700 | 0.3082085800 | 0.3979400090 |
|  | NCS | 0.3010299900 | - | 1.4913616940 | 0.4771212550 | 1.8920946030 | 0.3010299900 | - | 1.8750612630 | 0.3010299900 | 0.3010299960 |
|  | NCSF | 1.6334684560 | - | 3.0965624380 | 2.6273658570 | 2.3159703450 | 1.5563025010 | - | 2.5078558720 | 0.3010299900 | 0.3010299960 |
|  | NCSFL | 1.7634279940 | - | 3.2268575700 | 3.0203612830 | 2.5965970960 | 1.5563025010 | - | 2.6138418220 | 0.4771212550 | 0.6989700040 |
| DENT | NC | 2.3263358610 | - | 3.6800634270 | 3.2895889530 | 3.0759117610 | 2.6589648430 | 2.5301996980 | 2.6283889300 | 2.0681858620 | 1.6901960800 |
|  | H | 0.8450980400 | - | 1.5563025010 | 1.4313637640 | 1.2787536010 | 1.0791812460 | 1.0413926850 | 1.0000000000 | 0.8450980400 | 0.6989700040 |
|  | Hm | 0.6087297360 | - | 1.2374180560 | 1.0541026570 | 0.9388180710 | 0.3590219430 | 0.6068588740 | 0.7256394330 | 0.5131541580 | 0.5440680440 |
|  | NCS | 0.3010299900 | - | 1.7481880270 | 0.7781512500 | 1.6627578320 | 0.3010299900 | 0.3010299900 | 0.4771212550 | 0.3010299900 | 1.2552725050 |
|  | NCSF | 2.1461280360 | - | 3.0402066280 | 2.2900346110 | 2.5932860670 | 2.2304489210 | 2.0718820070 | 2.1986570870 | 1.7075701760 | 1.6901960800 |
|  | NCSFL | 2.1461280360 | - | 3.3577443250 | 2.9523080100 | 2.6522463410 | 2.2304489210 | 2.1818435880 | 2.2304489210 | 1.7075701760 | 1.6901960800 |
| EART | NC | 3.5144149210 | 3.0354297380 | 4.0030725960 | 3.2785249650 | 4.2564050180 | 3.0441476210 | 2.0681858620 | 3.3823773030 | 2.9978230810 | 2.8579352650 |
|  | H | 1.4623979980 | 1.1139433520 | 1.6334684560 | 1.4471580310 | 1.6434526760 | 1.3010299960 | 0.6989700040 | 1.3979400090 | 1.3010299960 | 1.2041199830 |
|  | Hm | 1.1572851260 | 0.7055379100 | 1.1458694500 | 0.9674090220 | 1.1376656240 | 0.9114964020 | 0.3358662010 | 0.9626554000 | 0.9048111680 | 0.8363403480 |
|  | NCS | 2.4668676200 | 0.6020599910 | 2.2329961100 | 2.2479732660 | 2.3222192950 | 1.2787536010 | 0.3010299900 | 1.5185139400 | 1.8388490910 | 1.4471580310 |
|  | NCSF | 2.8247764620 | 1.6812412370 | 3.2092468490 | 2.8280150640 | 2.7979596440 | 2.1072099700 | 0.6989700040 | 2.6127838570 | 2.2764618040 | 2.0681858620 |
|  | NCSFL | 3.2926990030 | 1.9731278540 | 3.3199384400 | 2.8350561020 | 3.1525940780 | 2.5340261060 | 1.3979400090 | 2.7693773260 | 2.6138418220 | 2.2430380490 |
| ECON | NC | 2.5943925500 | 1.0791812460 | 2.9360107960 | 2.9164539490 | 2.7084209000 | 2.0413926850 | 2.0934216850 | 2.7355989000 | 2.2068258760 | 1.8864907250 |
|  | H | 1.0413926850 | 0.4771212550 | 1.2787536010 | 1.2787536010 | 1.0791812460 | 0.6989700040 | 0.4771212550 | 1.1139433520 | 0.8450980400 | 0.6020599910 |
|  | Hm | 0.8750612630 | 0.4522976710 | 0.9769610160 | 0.9750332600 | 0.9790660930 | 0.5740312680 | 0.3357921020 | 0.8522766250 | 0.7781512500 | 0.6989700040 |
|  | NCS | 1.5185139400 | 0.3010299900 | 2.6884198220 | 1.9542425090 | 2.3222192950 | 1.2787536010 | 0.3010299900 | 1.6627578320 | 1.0413926850 | 1.3424226810 |
|  | NCSF | 2.4361626470 | 0.6020599910 | 2.7387805580 | 2.5899496010 | 2.5820633630 | 2.0413926850 | 2.0863598310 | 2.6222140230 | 2.1461280360 | 1.6720978580 |
|  | NCSFL | 2.4955443380 | 1.0791812460 | 2.8188854150 | 2.7323937600 | 2.6580113970 | 2.0413926850 | 2.0934216850 | 2.6434526760 | 2.1731862680 | 1.6901960800 |

**Table S3.** Continue.

| **FIELD** | **METRIC** | **COUNTRY** | | | | | | | | | |
| --- | --- | --- | --- | --- | --- | --- | --- | --- | --- | --- | --- |
|  |  | **ARG** | **BOL^*^** | **BRA** | **COL** | **CHI** | **ECU** | **PAR^*^** | **PER** | **URU** | **VEN** |
| ENER | NC | 2.9454685850 | 2.3424226810 | 3.4342494520 | 3.5864747790 | 3.5864747790 | 2.7634279940 | 2.1553360370 | 2.3096301670 | 3.0157787560 | 2.4623979980 |
|  | H | 1.2552725050 | 1.0000000000 | 1.4471580310 | 1.3979400090 | 1.3424226810 | 1.1760912590 | 0.9542425090 | 0.8450980400 | 1.3010299960 | 1.0000000000 |
|  | Hm | 1.0537185240 | 0.6215224710 | 1.1185848730 | 1.0817586690 | 1.0505086460 | 0.7522619130 | 0.5894708640 | 0.6643285190 | 0.7968200230 | 0.6197887580 |
|  | NCS | 1.9956351950 | 0.3010299900 | 1.9190780920 | 1.9684829490 | 1.7634279940 | 1.4471580310 | 0.3010299900 | 1.1139433520 | 0.3010299900 | 1.0791812460 |
|  | NCSF | 2.6009728960 | 1.9777236050 | 2.7050079590 | 3.0496056130 | 3.3062105080 | 2.4857214260 | 2.1492191130 | 2.3053513690 | 2.1875207210 | 2.0253058650 |
|  | NCSFL | 2.6830470380 | 1.9777236050 | 3.2365372610 | 3.2089785170 | 3.3096301670 | 2.4857214260 | 2.1492191130 | 2.3053513690 | 2.4771212550 | 2.0253058650 |
| ENGI | NC | 3.1430148000 | 3.0390173220 | 3.5094713520 | 3.5344068990 | 4.1550018360 | 3.3113299520 | 2.8744818180 | 3.3699576070 | 2.4593924880 | 3.0195316850 |
|  | H | 1.3222192950 | 1.1461280360 | 1.4913616940 | 1.4771212550 | 1.7323937600 | 1.3802112420 | 1.1760912590 | 1.4313637640 | 1.0413926850 | 1.2552725050 |
|  | Hm | 1.0654091340 | 0.5847887590 | 1.1753028620 | 1.1174028320 | 1.3665341290 | 0.9985499340 | 0.8553172050 | 1.2346859740 | 0.8450980400 | 0.9890046160 |
|  | NCS | 2.0644579890 | 0.3010299900 | 2.7395723440 | 2.4183012910 | 1.8260748030 | 1.5051499780 | 0.3010299900 | 1.9030899870 | 1.8750612630 | 1.7242758700 |
|  | NCSF | 2.9025467790 | 1.6020599910 | 3.0692980120 | 2.6739419990 | 3.4248816370 | 2.8155777480 | 2.2121876040 | 3.3153404770 | 2.2430380490 | 2.3926969530 |
|  | NCSFL | 3.0187004990 | 2.1958996520 | 3.2528530310 | 2.9084850190 | 3.8024316260 | 2.9965116720 | 2.3856062740 | 3.3410386320 | 2.3010299960 | 2.9425041060 |
| ENVI | NC | 3.4085791250 | 3.5899496010 | 3.7750276000 | 3.6760531250 | 3.4617985580 | 3.3255156630 | 2.2304489210 | 3.5453071160 | 3.2538224390 | 3.4479328660 |
|  | H | 1.4471580310 | 1.4623979980 | 1.6434526760 | 1.6720978580 | 1.4771212550 | 1.3617278360 | 0.7781512500 | 1.4913616940 | 1.3424226810 | 1.3222192950 |
|  | Hm | 1.1502579040 | 0.6674529530 | 1.3431344610 | 1.1468194240 | 1.2339022120 | 0.9948685670 | 0.5084808470 | 1.1264926540 | 1.0494648650 | 0.6275220800 |
|  | NCS | 2.0681858620 | 0.6989700040 | 2.9009130680 | 2.2121876040 | 1.9731278540 | 1.4149733480 | 0.7781512500 | 1.5682017240 | 1.5440680440 | 0.9030899870 |
|  | NCSF | 2.7723217070 | 2.4548448600 | 3.5332635170 | 2.9722028380 | 3.2211533220 | 2.7275412570 | 1.9030899870 | 3.0187004990 | 2.4502491080 | 2.2833012290 |
|  | NCSFL | 2.9680157140 | 2.5774918000 | 3.5365584430 | 3.5194341950 | 3.2322335210 | 2.7708520120 | 1.9030899870 | 3.0751818550 | 2.9708116110 | 2.2900346110 |
| HEAL | NC | 1.4623979980 | - | 2.5065050320 | 2.3242824550 | 2.7528164310 | 1.9777236050 | - | 1.3802112420 | 0.3010299900 | 1.1760912590 |
|  | H | 0.6020599910 | - | 1.0000000000 | 0.9030899870 | 1.1760912590 | 0.6020599910 | - | 0.6020599910 | 0.3010299900 | 0.6020599910 |
|  | Hm | 0.3400662210 | - | 0.7235302990 | 0.6170162350 | 0.7759680330 | 0.4983105540 | - | 0.5228787450 | 0.1995723550 | 0.3010299900 |
|  | NCS | 0.3010299900 | - | 1.7160033440 | 0.4771212550 | 0.4771212550 | 0.3010299900 | - | 0.3010299900 | 0.3010299900 | 1.0000000000 |
|  | NCSF | 1.1139433520 | - | 2.2201080880 | 2.1958996520 | 2.6739419990 | 1.9030899870 | - | 1.2552725050 | 0.3010299900 | 1.0000000000 |
|  | NCSFL | 1.2304489210 | - | 2.2201080880 | 2.1958996520 | 2.6757783420 | 1.9030899870 | - | 1.3802112420 | 0.3010299900 | 1.1760912590 |

**Table S3.** Continue.

| **FIELD** | **METRIC** | **COUNTRY** | | | | | | | | | |
| --- | --- | --- | --- | --- | --- | --- | --- | --- | --- | --- | --- |
|  |  | **ARG** | **BOL^*^** | **BRA** | **COL** | **CHI** | **ECU** | **PAR^*^** | **PER** | **URU** | **VEN** |
| IMMU | NC | 3.4171394100 | 2.5717088320 | 3.8418598100 | 3.2552725050 | 3.2314695900 | 3.1274287780 | 1.5440680440 | 3.2198463860 | 3.1705550590 | 2.9344984510 |
|  | H | 1.4471580310 | 1.1139433520 | 1.6020599910 | 1.4149733480 | 1.3979400090 | 1.2041199830 | 0.6020599910 | 1.4471580310 | 1.3802112420 | 1.0413926850 |
|  | Hm | 1.0546773040 | 0.5363179030 | 1.3028517460 | 0.9478772040 | 1.0427468060 | 0.6080503550 | 0.2228815550 | 0.9017096860 | 0.9106759290 | 0.6091961950 |
|  | NCS | 1.7481880270 | 0.3010299900 | 2.2068258760 | 1.5314789170 | 1.5185139400 | 0.3010299900 | 0.3010299900 | 1.3424226810 | 1.2787536010 | 1.1139433520 |
|  | NCSF | 3.0499928570 | 1.9684829490 | 3.2268575700 | 2.3765769570 | 2.7387805580 | 1.9084850190 | 0.8450980400 | 2.4955443380 | 2.3443922740 | 1.9822712330 |
|  | NCSFL | 3.2362852770 | 1.9684829490 | 3.5628873810 | 2.8853612200 | 3.0248959600 | 2.0293837780 | 0.8450980400 | 2.5998830720 | 2.5010592620 | 2.2304489210 |
| MATE | NC | 3.3998467130 | 2.3443922740 | 3.9641181430 | 3.4507108780 | 3.7498908410 | 3.2462523120 | - | 2.7234556720 | 3.0056094450 | 3.1271047980 |
|  | H | 1.4149733480 | 1.0000000000 | 1.6901960800 | 1.3617278360 | 1.5185139400 | 1.3617278360 | - | 1.1461280360 | 1.2787536010 | 1.3010299960 |
|  | Hm | 1.1731052960 | 0.5614421400 | 1.3430669130 | 0.9547451280 | 1.1153727380 | 0.9948685670 | - | 0.8850783840 | 1.0051881100 | 1.0940050220 |
|  | NCS | 2.4345689040 | 0.3010299900 | 2.0128372250 | 1.2787536010 | 2.4955443380 | 1.3979400090 | - | 1.6334684560 | 0.3010299900 | 0.6989700040 |
|  | NCSF | 2.9164539490 | 0.9030899870 | 3.1640552920 | 2.6532125140 | 3.6434526760 | 2.3873898260 | - | 2.5728716020 | 2.0334237550 | 2.7450747920 |
|  | NCSFL | 3.2674064190 | 0.9542425090 | 3.5581083020 | 2.7759743310 | 3.6434526760 | 2.8254261180 | - | 2.6776069530 | 2.6464037260 | 2.8142475960 |
| MATH | NC | 2.9503648540 | 1.8195439360 | 3.5869247080 | 2.9863237770 | 3.3051363190 | 2.6085260340 | 1.9190780920 | 2.3636119800 | 2.3873898260 | 2.4487063200 |
|  | H | 1.1139433520 | 0.7781512500 | 1.5440680440 | 1.2041199830 | 1.4313637640 | 1.0413926850 | 0.7781512500 | 1.0791812460 | 1.0000000000 | 1.0413926850 |
|  | Hm | 1.0731070980 | 0.4093694700 | 1.3013917570 | 0.9867717340 | 1.1538148640 | 0.9700367770 | 0.5185139400 | 1.0413926850 | 0.8553172050 | 0.9700367770 |
|  | NCS | 2.2944662260 | 0.3010299900 | 2.3856062740 | 1.7481880270 | 2.3783979010 | 1.9444826720 | 0.3010299900 | 2.1702617150 | 1.7481880270 | 1.9395192530 |
|  | NCSF | 2.4345689040 | 1.6434526760 | 3.1775365000 | 2.6946051990 | 2.9227254580 | 2.3201462860 | 1.5440680440 | 2.2121876040 | 1.9731278540 | 2.2329961100 |
|  | NCSFL | 2.5910646070 | 1.6434526760 | 3.3758464360 | 2.6954816760 | 2.9247959960 | 2.6063813650 | 1.5440680440 | 2.3636119800 | 2.1643528560 | 2.3521825180 |
| MEDI | NC | 4.4728587960 | 3.1271047980 | 4.7733182660 | 4.6384792730 | 4.6829659380 | 3.6832272060 | 3.4802944600 | 4.1266832630 | 4.4968328200 | 3.8788663370 |
|  | H | 1.7781512500 | 1.2787536010 | 1.8512583490 | 1.7160033440 | 1.5910646070 | 1.4771212550 | 1.4471580310 | 1.6532125140 | 1.3979400090 | 1.6020599910 |
|  | Hm | 1.2140243690 | 0.6935373740 | 1.3451878520 | 1.2633675580 | 1.1774125140 | 1.2542547420 | 0.9718372050 | 1.2041531120 | 1.0363568150 | 1.0017814340 |
|  | NCS | 2.0569048510 | 0.3010299960 | 2.4623979980 | 2.2304489210 | 2.5390760990 | 2.6766936100 | 1.3979400090 | 2.1760912590 | 2.4232458740 | 2.1271047980 |
|  | NCSF | 3.2550311630 | 2.2695129440 | 3.5890555310 | 3.3051363190 | 3.2467447100 | 3.2984163800 | 2.8785217960 | 2.9415114330 | 2.8864907250 | 2.7723217070 |
|  | NCSFL | 3.4339297660 | 2.2944662260 | 3.9222062770 | 3.6346787520 | 3.3186892700 | 3.3632358040 | 2.8932067530 | 3.2372923380 | 2.9675479760 | 2.8438554230 |

**Table S3.** Continue.

| **FIELD** | **METRIC** | **COUNTRY** | | | | | | | | | |
| --- | --- | --- | --- | --- | --- | --- | --- | --- | --- | --- | --- |
|  |  | **ARG** | **BOL^*^** | **BRA** | **COL** | **CHI** | **ECU** | **PAR^*^** | **PER** | **URU** | **VEN** |
| NEUR | NC | 3.6696887080 | - | 3.6697816150 | 3.2276296500 | 3.5389505620 | 2.5658478190 | - | 2.2600713880 | 3.2581581930 | 2.4941545940 |
|  | H | 1.6127838570 | - | 1.5563025010 | 1.4471580310 | 1.3802112420 | 1.1461280360 | - | 0.8450980400 | 1.2787536010 | 1.0000000000 |
|  | Hm | 1.1875856100 | - | 1.2065511830 | 0.9239714290 | 1.0508460340 | 0.7720771030 | - | 0.5106054880 | 0.9110114850 | 0.6253777880 |
|  | NCS | 1.6532125140 | - | 2.9749719940 | 0.3010299900 | 2.1303337680 | 0.3010299960 | - | 0.3010299900 | 1.1461280360 | 1.6901960800 |
|  | NCSF | 3.1956229440 | - | 3.1089031280 | 2.8260748030 | 2.9319661150 | 2.0934216850 | - | 0.3010299900 | 2.5092025220 | 1.8129133570 |
|  | NCSFL | 3.5475285760 | - | 3.3782161500 | 2.8500332580 | 3.2127201540 | 2.2855573090 | - | 1.4913616940 | 2.7291647900 | 2.1398790860 |
| NURS | NC | 2.2878017300 | - | 2.8524799940 | 2.5185139400 | 2.5728716020 | 1.4471580310 | - | 1.0413926850 | 2.7641761320 | 2.1238516410 |
|  | H | 0.7781512500 | - | 1.1760912590 | 1.0413926850 | 1.1461280360 | 0.4771212550 | - | 0.4771212550 | 1.1760912590 | 0.8450980400 |
|  | Hm | 0.3728243640 | - | 0.9502025320 | 0.6317818730 | 0.7034960890 | 0.3521825180 | - | 0.4771212550 | 0.7381350940 | 0.5294003520 |
|  | NCS | 0.3010299900 | - | 1.6532125140 | 1.1139433520 | 1.3979400090 | 0.3010299900 | - | 0.4771212550 | 0.7781512500 | 0.4771212550 |
|  | NCSF | 1.4313637640 | - | 2.4248816370 | 1.6127838570 | 2.1760912590 | 1.2552725050 | - | 0.8450980400 | 2.1931245980 | 2.0043213740 |
|  | NCSFL | 1.5314789170 | - | 2.6794278970 | 1.8388490910 | 2.1789769470 | 1.2552725050 | - | 0.8450980400 | 2.1931245980 | 2.0755469610 |
| PHAR | NC | 3.2697463730 | 2.3783979010 | 3.8017466190 | 3.0689276120 | 3.3912880490 | 2.6106601630 | 2.7118072290 | 2.6364878960 | 3.1577588860 | 2.3304137730 |
|  | H | 1.3222192950 | 1.0413926850 | 1.5563025010 | 1.3010299960 | 1.5051499780 | 1.0791812460 | 1.1760912590 | 1.0413926850 | 1.3802112420 | 0.9542425090 |
|  | Hm | 0.9952710570 | 0.5420616320 | 1.2708928480 | 0.8860414500 | 1.1926050610 | 0.7869653850 | 0.6380347440 | 0.6351920650 | 0.9715562380 | 0.6502956060 |
|  | NCS | 2.1789769470 | 0.3010299900 | 2.1846914310 | 1.6627578320 | 1.6434526760 | 0.3010299900 | 0.6989700040 | 0.3010299900 | 0.9542425090 | 0.4771212550 |
|  | NCSF | 2.5289167000 | 1.7075701760 | 2.9100905460 | 2.4329692910 | 3.1928461150 | 2.3521825180 | 1.7708520120 | 2.4487063200 | 2.0681858620 | 1.9242792860 |
|  | NCSFL | 2.9698816440 | 1.7075701760 | 3.3765769570 | 2.5809249760 | 3.2027606870 | 2.4785664960 | 2.2430380490 | 2.5717088320 | 2.5965970960 | 1.9395192530 |
| PHYS | NC | 4.7440738240 | 3.0707764630 | 5.1780440820 | 5.1780440820 | 5.1780440820 | 4.6071439360 | 2.5440680440 | 4.2302444990 | 3.0976043290 | 2.7466341990 |
|  | H | 2.0253058650 | 1.3222192950 | 2.1702617150 | 2.1702617150 | 2.1702617150 | 1.9731278540 | 1.0413926850 | 1.8573324960 | 1.2552725050 | 1.1139433520 |
|  | Hm | 1.0992200950 | 0.5816842320 | 1.3204924750 | 1.1482156990 | 1.2299310340 | 0.9606293080 | 0.6068588740 | 0.8482082630 | 1.0413926850 | 0.8151348170 |
|  | NCS | 2.2528530310 | 0.3010299960 | 2.6148972160 | 2.1205739310 | 2.7466341990 | 1.8864907250 | 0.8450980400 | 1.8388490910 | 1.8195439360 | 1.3010299960 |
|  | NCSF | 3.1908917170 | 1.7634279940 | 3.3087777740 | 3.0281644190 | 3.1159431770 | 2.5250448070 | 0.8450980400 | 2.5670263660 | 2.8068580300 | 2.2148438480 |
|  | NCSFL | 3.1942367490 | 1.9294189260 | 3.3961993470 | 3.2166935990 | 3.1914510140 | 2.5740312680 | 1.6901960800 | 2.5670263660 | 2.8779469520 | 2.3579348470 |

**Table S3.** Continue.

| **FIELD** | **METRIC** | **COUNTRY** | | | | | | | | | |
| --- | --- | --- | --- | --- | --- | --- | --- | --- | --- | --- | --- |
|  |  | **ARG** | **BOL^*^** | **BRA** | **COL** | **CHI** | **ECU** | **PAR^*^** | **PER** | **URU** | **VEN** |
| PSYC | NC | 2.6821450760 | 1.8920946030 | 3.6467956890 | 2.9242792860 | 3.0969100130 | 2.1613680020 | 1.7634279940 | 2.8344207040 | 3.4756711880 | 2.2355284470 |
|  | H | 1.1139433520 | 0.6020599910 | 1.5185139400 | 1.2041199830 | 1.2787536010 | 0.8450980400 | 0.6989700040 | 0.9542425090 | 1.0000000000 | 0.7781512500 |
|  | Hm | 0.8354892530 | 0.3252248050 | 1.2413415010 | 0.9878203310 | 0.8512583490 | 0.8750612630 | 0.4890204780 | 0.8356548370 | 0.6989700040 | 0.6532125140 |
|  | NCS | 1.5185139400 | 0.3010299900 | 2.2068258760 | 2.2227164710 | 1.6901960800 | 1.7481880270 | 0.7781512500 | 1.4771212550 | 1.2552725050 | 1.1760912590 |
|  | NCSF | 2.2988530760 | 0.3010299900 | 2.9772662120 | 2.7589118920 | 2.4487063200 | 1.8388490910 | 1.4149733480 | 1.9344984510 | 1.6812412370 | 1.4313637640 |
|  | NCSFL | 2.4857214260 | 0.3010299900 | 3.2382970680 | 2.7678976160 | 2.6095944090 | 1.8388490910 | 1.6532125140 | 2.1702617150 | 2.2718416070 | 1.6720978580 |
| SOCI | NC | 2.8920946030 | 2.2227164710 | 3.0149403500 | 3.3038437750 | 3.5217916500 | 2.8756399370 | 1.4913616940 | 2.7355989000 | 3.2741578490 | 2.9722028380 |
|  | H | 1.2041199830 | 1.0000000000 | 1.3010299960 | 1.3424226810 | 1.4313637640 | 1.0791812460 | 0.6989700040 | 1.1139433520 | 1.4149733480 | 1.1760912590 |
|  | Hm | 1.0211892990 | 0.6172999580 | 1.0837101730 | 1.1105897100 | 1.2126314410 | 0.9002015570 | 0.7403626890 | 0.8065278270 | 1.1461280360 | 1.1105897100 |
|  | NCS | 2.1461280360 | 1.4913616940 | 2.5171958980 | 2.2329961100 | 2.9324737650 | 1.9542425090 | 1.3979400090 | 1.8633228600 | 1.9684829490 | 2.2068258760 |
|  | NCSF | 2.5185139400 | 1.4913616940 | 2.6190933310 | 3.0503797560 | 3.3081373790 | 2.6720978580 | 1.4771212550 | 2.6222140230 | 3.1894903140 | 2.6117233080 |
|  | NCSFL | 2.7032913780 | 1.8061799740 | 2.8686444380 | 3.1085650240 | 3.5067755370 | 2.6730209070 | 1.4913616940 | 2.6434526760 | 3.2062860440 | 2.6117233080 |
| VETE | NC | 3.0132586650 | 1.3424226810 | 3.2902572690 | 2.7339992870 | 2.8579352650 | 2.1492191130 | 2.0000000000 | 2.6766936100 | 3.2773799750 | 1.9777236050 |
|  | H | 1.2787536010 | 0.6020599910 | 1.4313637640 | 1.1461280360 | 1.2304489210 | 0.9542425090 | 0.9030899870 | 1.0000000000 | 1.3010299960 | 0.6989700040 |
|  | Hm | 0.9846717850 | 0.1512676750 | 1.0314419830 | 0.8985423590 | 0.8500916740 | 0.5096504800 | 0.3638839180 | 0.6723316040 | 1.0497911750 | 0.4458635610 |
|  | NCS | 1.4471580310 | 0.3010299900 | 1.5563025010 | 0.4771212550 | 0.3010299900 | 0.3010299900 | 0.3010299900 | 0.3010299960 | 0.3010299900 | 0.3010299900 |
|  | NCSF | 2.3765769570 | 1.1760912590 | 2.4913616940 | 2.0211892990 | 2.2304489210 | 1.7558748560 | 1.4771212550 | 1.8633228600 | 2.1398790860 | 1.9777236050 |
|  | NCSFL | 2.6946051990 | 1.1760912590 | 3.1577588860 | 2.5693739100 | 2.7363965020 | 1.7558748560 | 1.4771212550 | 1.9731278540 | 2.9694159120 | 1.9777236050 |

* Bolivia and Paraguay has no representation in some fields for the 2011-2020 period; accordingly, no data is reported in some cases.

**Table S4.** Author count and percentage distribution of the South America-based scientists composite indicator in terms of the range quarters: Q4: [0.0-1.5[, Q3: [1.5-3.0[, Q2: [3.0-4.5[, and Q1: [4.5-6.0]. The data presented corresponds to the 2011-2020 period.

| **ARGENTINA** | | | | | | | | |
| --- | --- | --- | --- | --- | --- | --- | --- | --- |
| **Field** | **Count of Authors (#)** | | | **Percentage Distribution (%)** | | | | |
|  | **Q4** | **Q3** | **Q2** | **Q1** | **Q4** | **Q3** | **Q2** | **Q1** |
| AGRI | 2740 | 3452 | 298 | 12 | 42.1 | 53.1 | 4.6 | 0.2 |
| ARTS | 352 | 264 | 39 | 9 | 53.0 | 39.8 | 5.9 | 1.4 |
| BIOC | 1408 | 2241 | 179 | 16 | 36.6 | 58.3 | 4.7 | 0.4 |
| BUSI | 32 | 38 | 16 | 5 | 35.2 | 41.8 | 17.6 | 5.5 |
| CENG | 119 | 302 | 49 | 7 | 24.9 | 63.3 | 10.3 | 1.5 |
| CHEM | 408 | 853 | 159 | 11 | 28.5 | 59.6 | 11.1 | 0.8 |
| COMP | 535 | 389 | 21 | 9 | 56.1 | 40.8 | 2.2 | 0.9 |
| DECI | 1 | 2 | 0 | 0 | 33.3 | 66.7 | 0.0 | 0.0 |
| DENT | 28 | 9 | 0 | 0 | 75.7 | 24.3 | 0.0 | 0.0 |
| EART | 683 | 930 | 227 | 9 | 36.9 | 50.3 | 12.3 | 0.5 |
| ECON | 41 | 70 | 22 | 7 | 29.3 | 50.0 | 15.7 | 5.0 |
| ENER | 93 | 126 | 30 | 7 | 36.3 | 49.2 | 11.7 | 2.7 |
| ENGI | 578 | 442 | 60 | 11 | 53.0 | 40.5 | 5.5 | 1.0 |
| ENVI | 409 | 715 | 123 | 9 | 32.6 | 56.9 | 9.8 | 0.7 |
| HEAL | 17 | 25 | 9 | 0 | 33.3 | 49.0 | 17.6 | 0.0 |
| IMMU | 432 | 679 | 86 | 2 | 36.0 | 56.6 | 7.2 | 0.2 |
| MATE | 321 | 595 | 83 | 7 | 31.9 | 59.1 | 8.3 | 0.7 |
| MATH | 225 | 297 | 41 | 5 | 39.6 | 52.3 | 7.2 | 0.9 |
| MEDI | 7242 | 2277 | 175 | 11 | 74.6 | 23.5 | 1.8 | 0.1 |
| NEUR | 141 | 244 | 56 | 7 | 31.5 | 54.5 | 12.5 | 1.6 |
| NURS | 7 | 7 | 1 | 0 | 46.7 | 46.7 | 6.7 | 0.0 |
| PHAR | 221 | 248 | 36 | 7 | 43.2 | 48.4 | 7.0 | 1.4 |
| PHYS | 773 | 975 | 111 | 4 | 41.5 | 52.3 | 6.0 | 0.2 |
| PSYC | 125 | 158 | 22 | 5 | 40.3 | 51.0 | 7.1 | 1.6 |
| SOCI | 622 | 587 | 94 | 10 | 47.4 | 44.7 | 7.2 | 0.8 |
| VETE | 301 | 179 | 22 | 3 | 59.6 | 35.4 | 4.4 | 0.6 |
| AVERAGE | 686.7 | 619.4 | 75.3 | 6.7 |  |  |  |  |

**Table S4.** continue

| **BRAZIL** | | | | | | | | |
| --- | --- | --- | --- | --- | --- | --- | --- | --- |
| **Field** | **Count of Authors (#)** | | | **Percentage Distribution (%)** | | | | |
|  | **Q4** | **Q3** | **Q2** | **Q1** | **Q4** | **Q3** | **Q2** | **Q1** |
| AGRI | 18605 | 15635 | 1232 | 62 | 52.4 | 44.0 | 3.5 | 0.2 |
| ARTS | 703 | 313 | 47 | 14 | 65.3 | 29.1 | 4.4 | 1.3 |
| BIOC | 5093 | 7063 | 765 | 48 | 39.3 | 54.5 | 5.9 | 0.4 |
| BUSI | 1061 | 733 | 140 | 23 | 54.2 | 37.5 | 7.2 | 1.2 |
| CENG | 661 | 937 | 233 | 39 | 35.3 | 50.1 | 12.5 | 2.1 |
| CHEM | 2507 | 4268 | 796 | 54 | 32.9 | 56.0 | 10.4 | 0.7 |
| COMP | 4990 | 3738 | 277 | 27 | 55.2 | 41.4 | 3.1 | 0.3 |
| DECI | 80 | 79 | 32 | 7 | 40.4 | 39.9 | 16.2 | 3.5 |
| DENT | 2031 | 2155 | 431 | 28 | 43.7 | 46.4 | 9.3 | 0.6 |
| EART | 2111 | 1918 | 296 | 30 | 48.5 | 44.0 | 6.8 | 0.7 |
| ECON | 236 | 305 | 104 | 11 | 36.0 | 46.5 | 15.9 | 1.7 |
| ENER | 1028 | 964 | 200 | 27 | 46.3 | 43.4 | 9.0 | 1.2 |
| ENGI | 6879 | 4357 | 450 | 49 | 58.6 | 37.1 | 3.8 | 0.4 |
| ENVI | 2262 | 3137 | 457 | 54 | 38.3 | 53.1 | 7.7 | 0.9 |
| HEAL | 133 | 196 | 46 | 9 | 34.6 | 51.0 | 12.0 | 2.3 |
| IMMU | 1571 | 1854 | 275 | 22 | 42.2 | 49.8 | 7.4 | 0.6 |
| MATE | 2353 | 2903 | 371 | 48 | 41.5 | 51.2 | 6.5 | 0.8 |
| MATH | 948 | 1231 | 257 | 34 | 38.4 | 49.8 | 10.4 | 1.4 |
| MEDI | 29554 | 18272 | 1477 | 75 | 59.9 | 37.0 | 3.0 | 0.2 |
| NEUR | 808 | 1137 | 241 | 15 | 36.7 | 51.7 | 10.9 | 0.7 |
| NURS | 907 | 1415 | 296 | 20 | 34.4 | 53.6 | 11.2 | 0.8 |
| PHAR | 1560 | 2333 | 396 | 31 | 36.1 | 54.0 | 9.2 | 0.7 |
| PHYS | 3340 | 3334 | 450 | 48 | 46.6 | 46.5 | 6.3 | 0.7 |
| PSYC | 834 | 595 | 67 | 9 | 55.4 | 39.5 | 4.5 | 0.6 |
| SOCI | 2894 | 1713 | 193 | 28 | 59.9 | 35.5 | 4.0 | 0.6 |
| VETE | 3619 | 2255 | 271 | 15 | 58.8 | 36.6 | 4.4 | 0.2 |
| AVERAGE | 3721.8 | 3186.2 | 376.9 | 31.8 |  |  |  |  |

**Table S4.** continue

| **BOLIVIA** | | | | | | | | |
| --- | --- | --- | --- | --- | --- | --- | --- | --- |
| **Field** | **Count of Authors (#)** | | | **Percentage Distribution (%)** | | | | |
|  | **Q4** | **Q3** | **Q2** | **Q1** | **Q4** | **Q3** | **Q2** | **Q1** |
| AGRI | 75 | 47 | 4 | 0 | 59.5 | 37.3 | 3.2 | 0.0 |
| ARTS | 3 | 3 | 1 | 0 | 42.9 | 42.9 | 14.3 | 0.0 |
| BIOC | 12 | 10 | 3 | 0 | 48.0 | 40.0 | 12.0 | 0.0 |
| BUSI | 0 | 1 | 0 | 0 | 0.0 | 100.0 | 0.0 | 0.0 |
| CENG | 0 | 3 | 0 | 0 | 0.0 | 100.0 | 0.0 | 0.0 |
| CHEM | 3 | 1 | 1 | 0 | 60.0 | 20.0 | 20.0 | 0.0 |
| COMP | 5 | 4 | 1 | 0 | 50.0 | 40.0 | 10.0 | 0.0 |
| DECI | 0 | 0 | 0 | 0 | 0 | 0 | 0 | 0 |
| DENT | 0 | 0 | 0 | 0 | 0 | 0 | 0 | 0 |
| EART | 15 | 15 | 0 | 0 | 50.0 | 50.0 | 0.0 | 0.0 |
| ECON | 1 | 1 | 0 | 0 | 50.0 | 50.0 | 0.0 | 0.0 |
| ENER | 3 | 8 | 0 | 0 | 27.3 | 72.7 | 0.0 | 0.0 |
| ENGI | 2 | 2 | 1 | 0 | 40.0 | 40.0 | 20.0 | 0.0 |
| ENVI | 38 | 38 | 2 | 0 | 48.7 | 48.7 | 2.6 | 0.0 |
| HEAL | 1 | 1 | 0 | 0 | 50.0 | 50.0 | 0.0 | 0.0 |
| IMMU | 17 | 4 | 1 | 0 | 77.3 | 18.2 | 4.5 | 0.0 |
| MATE | 4 | 1 | 0 | 0 | 80.0 | 20.0 | 0.0 | 0.0 |
| MATH | 0 | 1 | 0 | 0 | 0.0 | 100.0 | 0.0 | 0.0 |
| MEDI | 126 | 31 | 4 | 0 | 78.3 | 19.3 | 2.5 | 0.0 |
| NEUR | 1 | 2 | 0 | 0 | 33.3 | 66.7 | 0.0 | 0.0 |
| NURS | 2 | 0 | 0 | 0 | 100.0 | 0.0 | 0.0 | 0.0 |
| PHAR | 6 | 6 | 0 | 0 | 50.0 | 50.0 | 0.0 | 0.0 |
| PHYS | 12 | 4 | 0 | 0 | 75.0 | 25.0 | 0.0 | 0.0 |
| PSYC | 0 | 1 | 0 | 0 | 0.0 | 100.0 | 0.0 | 0.0 |
| SOCI | 8 | 14 | 1 | 0 | 34.8 | 60.9 | 4.3 | 0.0 |
| VETE | 2 | 0 | 1 | 0 | 66.7 | 0.0 | 33.3 | 0.0 |
| AVERAGE | 12.9 | 7.6 | 0.8 | 0.0 |  |  |  |  |

**Table S4.** continue

| **COLOMBIA** | | | | | | | | |
| --- | --- | --- | --- | --- | --- | --- | --- | --- |
| **Field** | **Count of Authors (#)** | | | **Percentage Distribution (%)** | | | | |
|  | **Q4** | **Q3** | **Q2** | **Q1** | **Q4** | **Q3** | **Q2** | **Q1** |
| AGRI | 1174 | 860 | 95 | 13 | 54.8 | 40.1 | 4.4 | 0.6 |
| ARTS | 120 | 89 | 18 | 5 | 51.7 | 38.4 | 7.8 | 2.2 |
| BIOC | 245 | 244 | 38 | 4 | 46.1 | 46.0 | 7.2 | 0.8 |
| BUSI | 246 | 218 | 44 | 8 | 47.7 | 42.2 | 8.5 | 1.6 |
| CENG | 137 | 123 | 36 | 7 | 45.2 | 40.6 | 11.9 | 2.3 |
| CHEM | 154 | 242 | 62 | 5 | 33.3 | 52.3 | 13.4 | 1.1 |
| COMP | 738 | 575 | 54 | 12 | 0.9 | 3.9 | 41.7 | 53.5 |
| DECI | 8 | 9 | 5 | 1 | 34.8 | 39.1 | 21.7 | 4.3 |
| DENT | 53 | 27 | 6 | 0 | 61.6 | 31.4 | 7.0 | 0.0 |
| EART | 203 | 175 | 38 | 4 | 48.3 | 41.7 | 9.0 | 1.0 |
| ECON | 73 | 98 | 23 | 6 | 36.5 | 49.0 | 11.5 | 3.0 |
| ENER | 162 | 205 | 51 | 7 | 38.1 | 48.2 | 12.0 | 1.6 |
| ENGI | 824 | 678 | 78 | 12 | 51.8 | 42.6 | 4.9 | 0.8 |
| ENVI | 158 | 295 | 49 | 9 | 30.9 | 57.7 | 9.6 | 1.8 |
| HEAL | 12 | 9 | 3 | 0 | 50.0 | 37.5 | 12.5 | 0.0 |
| IMMU | 90 | 86 | 23 | 4 | 44.3 | 42.4 | 11.3 | 2.0 |
| MATE | 155 | 214 | 23 | 0 | 39.5 | 54.6 | 5.9 | 0.0 |
| MATH | 181 | 196 | 26 | 1 | 44.8 | 48.5 | 6.4 | 0.2 |
| MEDI | 3629 | 1264 | 112 | 8 | 72.4 | 25.2 | 2.2 | 0.2 |
| NEUR | 16 | 27 | 8 | 3 | 29.6 | 50.0 | 14.8 | 5.6 |
| NURS | 24 | 39 | 7 | 0 | 34.3 | 55.7 | 10.0 | 0.0 |
| PHAR | 123 | 83 | 12 | 2 | 55.9 | 37.7 | 5.5 | 0.9 |
| PHYS | 581 | 354 | 37 | 3 | 59.6 | 36.3 | 3.8 | 0.3 |
| PSYC | 84 | 92 | 10 | 3 | 44.4 | 48.7 | 5.3 | 1.6 |
| SOCI | 385 | 319 | 51 | 11 | 50.3 | 41.6 | 6.7 | 1.4 |
| VETE | 98 | 42 | 10 | 0 | 65.3 | 28.0 | 6.7 | 0.0 |
| AVERAGE | 372.0 | 252.4 | 35.3 | 4.9 |  |  |  |  |

**Table S4.** continue

| **CHILE** | | | | | | | | |
| --- | --- | --- | --- | --- | --- | --- | --- | --- |
| **Field** | **Count of Authors (#)** | | | **Percentage Distribution (%)** | | | | |
|  | **Q4** | **Q3** | **Q2** | **Q1** | **Q4** | **Q3** | **Q2** | **Q1** |
| AGRI | 1258 | 1418 | 209 | 10 | 43.5 | 49.0 | 7.2 | 0.3 |
| ARTS | 363 | 295 | 47 | 10 | 50.8 | 41.3 | 6.6 | 1.4 |
| BIOC | 536 | 1032 | 147 | 7 | 31.1 | 59.9 | 8.5 | 0.4 |
| BUSI | 44 | 73 | 26 | 9 | 28.9 | 48.0 | 17.1 | 5.9 |
| CENG | 37 | 65 | 26 | 4 | 28.0 | 49.2 | 19.7 | 3.0 |
| CHEM | 287 | 514 | 107 | 6 | 31.4 | 56.2 | 11.7 | 0.7 |
| COMP | 381 | 449 | 68 | 9 | 42.0 | 49.5 | 7.5 | 1.0 |
| DECI | 4 | 12 | 8 | 2 | 15.4 | 46.2 | 30.8 | 7.7 |
| DENT | 74 | 74 | 18 | 1 | 44.3 | 44.3 | 10.8 | 0.6 |
| EART | 348 | 457 | 117 | 11 | 37.3 | 49.0 | 12.5 | 1.2 |
| ECON | 72 | 95 | 52 | 9 | 31.6 | 41.7 | 22.8 | 3.9 |
| ENER | 55 | 71 | 46 | 7 | 30.7 | 39.7 | 25.7 | 3.9 |
| ENGI | 430 | 452 | 94 | 13 | 43.5 | 45.7 | 9.5 | 1.3 |
| ENVI | 248 | 411 | 102 | 8 | 32.2 | 53.4 | 13.3 | 1.0 |
| HEAL | 15 | 26 | 10 | 1 | 28.8 | 50.0 | 19.2 | 1.9 |
| IMMU | 110 | 177 | 30 | 1 | 34.6 | 55.7 | 9.4 | 0.3 |
| MATE | 174 | 316 | 47 | 5 | 32.1 | 58.3 | 8.7 | 0.9 |
| MATH | 159 | 360 | 92 | 4 | 25.9 | 58.5 | 15.0 | 0.7 |
| MEDI | 4240 | 1547 | 142 | 10 | 71.4 | 26.0 | 2.4 | 0.2 |
| NEUR | 69 | 115 | 39 | 6 | 30.1 | 50.2 | 17.0 | 2.6 |
| NURS | 64 | 84 | 16 | 1 | 38.8 | 50.9 | 9.7 | 0.6 |
| PHAR | 87 | 85 | 16 | 4 | 45.3 | 44.3 | 8.3 | 2.1 |
| PHYS | 485 | 816 | 214 | 6 | 31.9 | 53.6 | 14.1 | 0.4 |
| PSYC | 100 | 169 | 49 | 7 | 30.8 | 52.0 | 15.1 | 2.2 |
| SOCI | 677 | 822 | 162 | 22 | 40.2 | 48.8 | 9.6 | 1.3 |
| VETE | 92 | 81 | 12 | 0 | 49.7 | 43.8 | 6.5 | 0.0 |
| AVERAGE | 400.3 | 385.2 | 72.9 | 6.7 |  |  |  |  |

**Table S4.** continue

| **PARAGUAY** | | | | | | | | |
| --- | --- | --- | --- | --- | --- | --- | --- | --- |
| **Field** | **Count of Authors (#)** | | | **Percentage Distribution (%)** | | | | |
|  | **Q4** | **Q3** | **Q2** | **Q1** | **Q4** | **Q3** | **Q2** | **Q1** |
| AGRI | 35 | 21 | 0 | 0 | 62.5 | 37.5 | 0.0 | 0.0 |
| ARTS | 1 | 0 | 0 | 0 | 100.0 | 0.0 | 0.0 | 0.0 |
| BIOC | 5 | 8 | 0 | 0 | 38.5 | 61.5 | 0.0 | 0.0 |
| BUSI | 2 | 0 | 0 | 0 | 100.0 | 0.0 | 0.0 | 0.0 |
| CENG | 0 | 0 | 0 | 0 | 0.0 | 0.0 | 0.0 | 0.0 |
| CHEM | 2 | 0 | 0 | 0 | 100.0 | 0.0 | 0.0 | 0.0 |
| COMP | 24 | 18 | 0 | 0 | 57.1 | 42.9 | 0.0 | 0.0 |
| DECI | 0 | 0 | 0 | 0 | 0 | 0 | 0 | 0 |
| DENT | 1 | 1 | 1 | 0 | 33.3 | 33.3 | 33.3 | 0.0 |
| EART | 1 | 1 | 0 | 0 | 50.0 | 50.0 | 0.0 | 0.0 |
| ECON | 0 | 1 | 0 | 0 | 0.0 | 100.0 | 0.0 | 0.0 |
| ENER | 2 | 1 | 1 | 0 | 50.0 | 25.0 | 25.0 | 0.0 |
| ENGI | 12 | 9 | 3 | 0 | 50.0 | 37.5 | 12.5 | 0.0 |
| ENVI | 6 | 3 | 0 | 0 | 66.7 | 33.3 | 0.0 | 0.0 |
| HEAL | 0 | 0 | 0 | 0 | 0 | 0 | 0 | 0 |
| IMMU | 13 | 0 | 0 | 0 | 100.0 | 0.0 | 0.0 | 0.0 |
| MATE | 0 | 0 | 0 | 0 | 0 | 0 | 0 | 0 |
| MATH | 1 | 1 | 1 | 0 | 33.3 | 33.3 | 33.3 | 0.0 |
| MEDI | 144 | 28 | 6 | 0 | 80.9 | 15.7 | 3.4 | 0.0 |
| NEUR | 0 | 0 | 0 | 0 | 0 | 0 | 0 | 0 |
| NURS | 1 | 0 | 0 | 0 | 100.0 | 0.0 | 0.0 | 0.0 |
| PHAR | 13 | 12 | 2 | 0 | 48.1 | 44.4 | 7.4 | 0.0 |
| PHYS | 6 | 2 | 1 | 0 | 66.7 | 22.2 | 11.1 | 0.0 |
| PSYC | 2 | 2 | 0 | 0 | 50.0 | 50.0 | 0.0 | 0.0 |
| SOCI | 2 | 2 | 0 | 0 | 50.0 | 50.0 | 0.0 | 0.0 |
| VETE | 2 | 1 | 0 | 0 | 66.7 | 33.3 | 0.0 | 0.0 |
| AVERAGE | 10.6 | 4.3 | 0.6 | 0.0 |  |  |  |  |

**Table S4.** continue

| **PERU** | | | | | | | | |
| --- | --- | --- | --- | --- | --- | --- | --- | --- |
| **Field** | **Count of Authors (#)** | | | **Percentage Distribution (%)** | | | | |
|  | **Q4** | **Q3** | **Q2** | **Q1** | **Q4** | **Q3** | **Q2** | **Q1** |
| AGRI | 390 | 234 | 28 | 2 | 59.6 | 35.8 | 4.3 | 0.3 |
| ARTS | 20 | 24 | 4 | 1 | 40.8 | 49.0 | 8.2 | 2.0 |
| BIOC | 59 | 59 | 6 | 1 | 47.2 | 47.2 | 4.8 | 0.8 |
| BUSI | 28 | 31 | 9 | 6 | 37.8 | 41.9 | 12.2 | 8.1 |
| CENG | 4 | 3 | 0 | 0 | 57.1 | 42.9 | 0.0 | 0.0 |
| CHEM | 26 | 19 | 2 | 0 | 55.3 | 40.4 | 4.3 | 0.0 |
| COMP | 279 | 103 | 7 | 1 | 71.5 | 26.4 | 1.8 | 0.3 |
| DECI | 0 | 2 | 0 | 1 | 0.0 | 66.7 | 0.0 | 33.3 |
| DENT | 46 | 23 | 3 | 0 | 63.9 | 31.9 | 4.2 | 0.0 |
| EART | 66 | 69 | 9 | 1 | 45.5 | 47.6 | 6.2 | 0.7 |
| ECON | 9 | 15 | 12 | 3 | 23.1 | 38.5 | 30.8 | 7.7 |
| ENER | 28 | 8 | 0 | 1 | 75.7 | 21.6 | 0.0 | 2.7 |
| ENGI | 202 | 98 | 8 | 6 | 64.3 | 31.2 | 2.5 | 1.9 |
| ENVI | 74 | 53 | 11 | 1 | 53.2 | 38.1 | 7.9 | 0.7 |
| HEAL | 3 | 2 | 0 | 0 | 60.0 | 40.0 | 0.0 | 0.0 |
| IMMU | 46 | 23 | 3 | 0 | 63.9 | 31.9 | 4.2 | 0.0 |
| MATE | 28 | 25 | 4 | 1 | 48.3 | 43.1 | 6.9 | 1.7 |
| MATH | 19 | 11 | 4 | 1 | 54.3 | 31.4 | 11.4 | 2.9 |
| MEDI | 1332 | 433 | 34 | 4 | 73.9 | 24.0 | 1.9 | 0.2 |
| NEUR | 3 | 2 | 0 | 0 | 60.0 | 40.0 | 0.0 | 0.0 |
| NURS | 3 | 2 | 0 | 0 | 60.0 | 40.0 | 0.0 | 0.0 |
| PHAR | 29 | 19 | 2 | 0 | 58.0 | 38.0 | 4.0 | 0.0 |
| PHYS | 94 | 39 | 5 | 3 | 66.7 | 27.7 | 3.5 | 2.1 |
| PSYC | 21 | 20 | 4 | 1 | 45.7 | 43.5 | 8.7 | 2.2 |
| SOCI | 148 | 91 | 19 | 4 | 56.5 | 34.7 | 7.3 | 1.5 |
| VETE | 168 | 60 | 1 | 0 | 73.4 | 26.2 | 0.4 | 0.0 |
| AVERAGE | 120.2 | 56.5 | 6.7 | 1.5 |  |  |  |  |

**Table S4.** continue

| **URUGUAY** | | | | | | | | |
| --- | --- | --- | --- | --- | --- | --- | --- | --- |
| **Field** | **Count of Authors (#)** | | | **Percentage Distribution (%)** | | | | |
|  | **Q4** | **Q3** | **Q2** | **Q1** | **Q4** | **Q3** | **Q2** | **Q1** |
| AGRI | 278 | 334 | 32 | 2 | 43.0 | 51.7 | 5.0 | 0.3 |
| ARTS | 16 | 13 | 2 | 0 | 51.6 | 41.9 | 6.5 | 0.0 |
| BIOC | 112 | 229 | 21 | 2 | 30.8 | 62.9 | 5.8 | 0.5 |
| BUSI | 4 | 10 | 3 | 0 | 23.5 | 58.8 | 17.6 | 0.0 |
| CENG | 3 | 11 | 1 | 0 | 20.0 | 73.3 | 6.7 | 0.0 |
| CHEM | 24 | 84 | 6 | 2 | 20.7 | 72.4 | 5.2 | 1.7 |
| COMP | 73 | 80 | 6 | 2 | 45.3 | 49.7 | 3.7 | 1.2 |
| DECI | 2 | 0 | 0 | 0 | 100.0 | 0.0 | 0.0 | 0.0 |
| DENT | 3 | 1 | 0 | 0 | 75.0 | 25.0 | 0.0 | 0.0 |
| EART | 22 | 41 | 12 | 1 | 28.9 | 53.9 | 15.8 | 1.3 |
| ECON | 3 | 17 | 5 | 1 | 11.5 | 65.4 | 19.2 | 3.8 |
| ENER | 18 | 22 | 4 | 0 | 40.9 | 50.0 | 9.1 | 0.0 |
| ENGI | 59 | 34 | 1 | 1 | 62.1 | 35.8 | 1.1 | 1.1 |
| ENVI | 25 | 63 | 12 | 2 | 24.5 | 61.8 | 11.8 | 2.0 |
| HEAL | 0 | 0 | 0 | 0 | 0 | 0 | 0 | 0 |
| IMMU | 58 | 58 | 9 | 0 | 46.4 | 46.4 | 7.2 | 0.0 |
| MATE | 15 | 16 | 2 | 1 | 44.1 | 47.1 | 5.9 | 2.9 |
| MATH | 28 | 30 | 8 | 2 | 41.2 | 44.1 | 11.8 | 2.9 |
| MEDI | 501 | 179 | 14 | 1 | 72.1 | 25.8 | 2.0 | 0.1 |
| NEUR | 17 | 33 | 11 | 0 | 27.9 | 54.1 | 18.0 | 0.0 |
| NURS | 1 | 1 | 0 | 1 | 33.3 | 33.3 | 0.0 | 33.3 |
| PHAR | 23 | 33 | 5 | 0 | 37.7 | 54.1 | 8.2 | 0.0 |
| PHYS | 33 | 45 | 9 | 0 | 37.9 | 51.7 | 10.3 | 0.0 |
| PSYC | 9 | 10 | 3 | 1 | 39.1 | 43.5 | 13.0 | 4.3 |
| SOCI | 35 | 50 | 10 | 2 | 36.1 | 51.5 | 10.3 | 2.1 |
| VETE | 37 | 47 | 5 | 1 | 41.1 | 52.2 | 5.6 | 1.1 |
| AVERAGE | 53.8 | 55.4 | 7.0 | 0.8 |  |  |  |  |

**Table S4.** continue

| **VENEZUELA** | | | | | | | | |
| --- | --- | --- | --- | --- | --- | --- | --- | --- |
| **Field** | **Count of Authors (#)** | | | **Percentage Distribution (%)** | | | | |
|  | **Q4** | **Q3** | **Q2** | **Q1** | **Q4** | **Q3** | **Q2** | **Q1** |
| AGRI | 637 | 166 | 13 | 1 | 78.0 | 20.3 | 1.6 | 0.1 |
| ARTS | 30 | 8 | 2 | 2 | 71.4 | 19.0 | 4.8 | 4.8 |
| BIOC | 123 | 44 | 3 | 1 | 71.9 | 25.7 | 1.8 | 0.6 |
| BUSI | 23 | 2 | 2 | 0 | 85.2 | 7.4 | 7.4 | 0.0 |
| CENG | 52 | 27 | 3 | 0 | 63.4 | 32.9 | 3.7 | 0.0 |
| CHEM | 154 | 75 | 10 | 0 | 64.4 | 31.4 | 4.2 | 0.0 |
| COMP | 91 | 36 | 4 | 0 | 69.5 | 27.5 | 3.1 | 0.0 |
| DECI | 1 | 0 | 0 | 0 | 100.0 | 0.0 | 0.0 | 0.0 |
| DENT | 7 | 1 | 1 | 0 | 77.8 | 11.1 | 11.1 | 0.0 |
| EART | 74 | 29 | 5 | 0 | 68.5 | 26.9 | 4.6 | 0.0 |
| ECON | 1 | 4 | 3 | 0 | 12.5 | 50.0 | 37.5 | 0.0 |
| ENER | 40 | 20 | 3 | 0 | 63.5 | 31.7 | 4.8 | 0.0 |
| ENGI | 214 | 40 | 9 | 0 | 81.4 | 15.2 | 3.4 | 0.0 |
| ENVI | 60 | 20 | 4 | 0 | 71.4 | 23.8 | 4.8 | 0.0 |
| HEAL | 0 | 0 | 0 | 1 | 0.0 | 0.0 | 0.0 | 100.0 |
| IMMU | 73 | 36 | 1 | 0 | 66.4 | 32.7 | 0.9 | 0.0 |
| MATE | 135 | 55 | 6 | 1 | 68.5 | 27.9 | 3.0 | 0.5 |
| MATH | 65 | 30 | 4 | 0 | 65.7 | 30.3 | 4.0 | 0.0 |
| MEDI | 1283 | 195 | 13 | 2 | 85.9 | 13.1 | 0.9 | 0.1 |
| NEUR | 15 | 9 | 2 | 1 | 55.6 | 33.3 | 7.4 | 3.7 |
| NURS | 18 | 10 | 3 | 0 | 58.1 | 32.3 | 9.7 | 0.0 |
| PHAR | 51 | 39 | 0 | 0 | 56.7 | 43.3 | 0.0 | 0.0 |
| PHYS | 113 | 55 | 3 | 1 | 65.7 | 32.0 | 1.7 | 0.6 |
| PSYC | 11 | 6 | 0 | 0 | 64.7 | 35.3 | 0.0 | 0.0 |
| SOCI | 110 | 26 | 7 | 3 | 75.3 | 17.8 | 4.8 | 2.1 |
| VETE | 150 | 20 | 0 | 0 | 88.2 | 11.8 | 0.0 | 0.0 |
| AVERAGE | 135.8 | 36.7 | 3.9 | 0.5 |  |  |  |  |

**Table S5.** Author count and percentage distribution of the South America-based scientists composite indicator in terms of the range quarters: Q4: [0.0-1.5[. Q3: [1.5-3.0[. Q2: [3.0-4.5[. and Q1: [4.5-6.0]. The data presented corresponds to the 2011-2020 period and the A1 authors dataset.

| **ARGENTINA** | | | | | | | | |
| --- | --- | --- | --- | --- | --- | --- | --- | --- |
| **Field** | **Count of Authors (#)** | | | **Percentage Distribution (%)** | | | | |
|  | **Q4** | **Q3** | **Q2** | **Q1** | **Q4** | **Q3** | **Q2** | **Q1** |
| AGRI | 2780 | 3557 | 316 | 14 | 41.70 | 53.35 | 4.74 | 0.21 |
| ARTS | 366 | 280 | 42 | 10 | 52.44 | 40.11 | 6.02 | 1.43 |
| BIOC | 1421 | 2281 | 186 | 16 | 36.40 | 58.43 | 4.76 | 0.41 |
| BUSI | 36 | 42 | 20 | 5 | 34.95 | 40.78 | 19.42 | 4.85 |
| CENG | 120 | 303 | 51 | 7 | 24.95 | 62.99 | 10.60 | 1.46 |
| CHEM | 413 | 882 | 168 | 11 | 28.02 | 59.84 | 11.40 | 0.75 |
| COMP | 568 | 425 | 25 | 9 | 55.31 | 41.38 | 2.43 | 0.88 |
| DECI | 1 | 2 | 0 | 0 | 33.33 | 66.67 | 0.00 | 0.00 |
| DENT | 32 | 11 | 2 | 0 | 71.11 | 24.44 | 4.44 | 0.00 |
| EART | 687 | 956 | 240 | 10 | 36.29 | 50.50 | 12.68 | 0.53 |
| ECON | 41 | 72 | 24 | 7 | 28.47 | 50.00 | 16.67 | 4.86 |
| ENER | 94 | 132 | 35 | 7 | 35.07 | 49.25 | 13.06 | 2.61 |
| ENGI | 594 | 457 | 63 | 11 | 52.80 | 40.62 | 5.60 | 0.98 |
| ENVI | 411 | 740 | 129 | 9 | 31.89 | 57.41 | 10.01 | 0.70 |
| HEAL | 17 | 26 | 9 | 0 | 32.69 | 50.00 | 17.31 | 0.00 |
| IMMU | 436 | 689 | 90 | 2 | 35.83 | 56.61 | 7.40 | 0.16 |
| MATE | 324 | 615 | 87 | 7 | 31.36 | 59.54 | 8.42 | 0.68 |
| MATH | 231 | 313 | 50 | 5 | 38.56 | 52.25 | 8.35 | 0.83 |
| MEDI | 7351 | 2399 | 202 | 12 | 73.78 | 24.08 | 2.03 | 0.12 |
| NEUR | 143 | 252 | 64 | 10 | 30.49 | 53.73 | 13.65 | 2.13 |
| NURS | 8 | 7 | 1 | 0 | 50.00 | 43.75 | 6.25 | 0.00 |
| PHAR | 225 | 253 | 38 | 7 | 43.02 | 48.37 | 7.27 | 1.34 |
| PHYS | 790 | 1035 | 124 | 4 | 40.45 | 53.00 | 6.35 | 0.20 |
| PSYC | 126 | 161 | 26 | 6 | 39.50 | 50.47 | 8.15 | 1.88 |
| SOCI | 650 | 633 | 107 | 10 | 46.43 | 45.21 | 7.64 | 0.71 |
| VETE | 306 | 185 | 25 | 3 | 58.96 | 35.65 | 4.82 | 0.58 |
| AVERAGE | 698.9 | 642.6 | 81.7 | 7.0 |  |  |  |  |

**Table S5.** continue

| **BRAZIL** | | | | | | | | |
| --- | --- | --- | --- | --- | --- | --- | --- | --- |
| **Field** | **Count of Authors (#)** | | | **Percentage Distribution (%)** | | | | |
|  | **Q4** | **Q3** | **Q2** | **Q1** | **Q4** | **Q3** | **Q2** | **Q1** |
| AGRI | 18725 | 15887 | 1263 | 64 | 52.10 | 44.21 | 3.51 | 0.18 |
| ARTS | 717 | 327 | 48 | 14 | 64.83 | 29.57 | 4.34 | 1.27 |
| BIOC | 5107 | 7152 | 784 | 49 | 39.01 | 54.63 | 5.99 | 0.37 |
| BUSI | 1069 | 744 | 146 | 23 | 53.94 | 37.54 | 7.37 | 1.16 |
| CENG | 663 | 948 | 238 | 41 | 35.08 | 50.16 | 12.59 | 2.17 |
| CHEM | 2513 | 4310 | 810 | 57 | 32.68 | 56.05 | 10.53 | 0.74 |
| COMP | 5038 | 3802 | 279 | 27 | 55.08 | 41.57 | 3.05 | 0.30 |
| DECI | 80 | 80 | 32 | 7 | 40.20 | 40.20 | 16.08 | 3.52 |
| DENT | 2043 | 2183 | 443 | 28 | 43.50 | 46.48 | 9.43 | 0.60 |
| EART | 2126 | 1976 | 308 | 30 | 47.88 | 44.50 | 6.94 | 0.68 |
| ECON | 238 | 310 | 107 | 11 | 35.74 | 46.55 | 16.07 | 1.65 |
| ENER | 1040 | 982 | 210 | 28 | 46.02 | 43.45 | 9.29 | 1.24 |
| ENGI | 6928 | 4434 | 460 | 50 | 58.36 | 37.35 | 3.87 | 0.42 |
| ENVI | 2280 | 3181 | 469 | 55 | 38.10 | 53.15 | 7.84 | 0.92 |
| HEAL | 134 | 199 | 47 | 9 | 34.45 | 51.16 | 12.08 | 2.31 |
| IMMU | 1574 | 1880 | 283 | 22 | 41.87 | 50.01 | 7.53 | 0.59 |
| MATE | 2368 | 2953 | 376 | 48 | 41.22 | 51.40 | 6.54 | 0.84 |
| MATH | 968 | 1273 | 268 | 35 | 38.05 | 50.04 | 10.53 | 1.38 |
| MEDI | 29692 | 18479 | 1510 | 78 | 59.67 | 37.14 | 3.03 | 0.16 |
| NEUR | 809 | 1147 | 244 | 15 | 36.52 | 51.78 | 11.02 | 0.68 |
| NURS | 909 | 1424 | 298 | 20 | 34.29 | 53.72 | 11.24 | 0.75 |
| PHAR | 1562 | 2345 | 404 | 32 | 35.97 | 53.99 | 9.30 | 0.74 |
| PHYS | 3392 | 3490 | 485 | 50 | 45.73 | 47.05 | 6.54 | 0.67 |
| PSYC | 839 | 599 | 68 | 9 | 55.38 | 39.54 | 4.49 | 0.59 |
| SOCI | 2935 | 1762 | 200 | 29 | 59.58 | 35.77 | 4.06 | 0.59 |
| VETE | 3640 | 2288 | 274 | 16 | 58.54 | 36.80 | 4.41 | 0.26 |
| AVERAGE | 3745.7 | 3236.7 | 386.7 | 32.6 |  |  |  |  |

**Table S5.** continue

| **BOLIVIA** | | | | | | | | |
| --- | --- | --- | --- | --- | --- | --- | --- | --- |
| **Field** | **Count of Authors (#)** | | | **Percentage Distribution (%)** | | | | |
|  | **Q4** | **Q3** | **Q2** | **Q1** | **Q4** | **Q3** | **Q2** | **Q1** |
| AGRI | 92 | 63 | 5 | 0 | 57.50 | 39.38 | 3.13 | 0.00 |
| ARTS | 3 | 3 | 1 | 0 | 42.86 | 42.86 | 14.29 | 0.00 |
| BIOC | 12 | 11 | 4 | 0 | 44.44 | 40.74 | 14.81 | 0.00 |
| BUSI | 0 | 1 | 0 | 0 | 0.00 | 100.00 | 0.00 | 0.00 |
| CENG | 0 | 3 | 0 | 0 | 0.00 | 100.00 | 0.00 | 0.00 |
| CHEM | 3 | 1 | 1 | 0 | 60.00 | 20.00 | 20.00 | 0.00 |
| COMP | 5 | 6 | 1 | 0 | 41.67 | 50.00 | 8.33 | 0.00 |
| DECI | 0 | 0 | 0 | 0 | 0.00 | 0.00 | 0.00 | 0.00 |
| DENT | 0 | 0 | 1 | 0 | 0.00 | 0.00 | 100.00 | 0.00 |
| EART | 16 | 19 | 4 | 0 | 41.03 | 48.72 | 10.26 | 0.00 |
| ECON | 1 | 2 | 0 | 0 | 33.33 | 66.67 | 0.00 | 0.00 |
| ENER | 3 | 9 | 2 | 0 | 21.43 | 64.29 | 14.29 | 0.00 |
| ENGI | 2 | 4 | 1 | 0 | 28.57 | 57.14 | 14.29 | 0.00 |
| ENVI | 39 | 42 | 5 | 0 | 45.35 | 48.84 | 5.81 | 0.00 |
| HEAL | 1 | 1 | 0 | 0 | 50.00 | 50.00 | 0.00 | 0.00 |
| IMMU | 17 | 4 | 2 | 0 | 73.91 | 17.39 | 8.70 | 0.00 |
| MATE | 4 | 1 | 0 | 0 | 80.00 | 20.00 | 0.00 | 0.00 |
| MATH | 1 | 1 | 0 | 0 | 50.00 | 50.00 | 0.00 | 0.00 |
| MEDI | 135 | 38 | 8 | 1 | 74.18 | 20.88 | 4.40 | 0.55 |
| NEUR | 1 | 2 | 1 | 0 | 25.00 | 50.00 | 25.00 | 0.00 |
| NURS | 2 | 0 | 0 | 0 | 100.00 | 0.00 | 0.00 | 0.00 |
| PHAR | 6 | 6 | 0 | 0 | 50.00 | 50.00 | 0.00 | 0.00 |
| PHYS | 12 | 5 | 1 | 0 | 66.67 | 27.78 | 5.56 | 0.00 |
| PSYC | 0 | 1 | 1 | 0 | 0.00 | 50.00 | 50.00 | 0.00 |
| SOCI | 10 | 18 | 1 | 0 | 34.48 | 62.07 | 3.45 | 0.00 |
| VETE | 4 | 0 | 1 | 0 | 80.00 | 0.00 | 20.00 | 0.00 |
| AVERAGE | 14.2 | 9.3 | 1.5 | 0.0 |  |  |  |  |

**Table S5.** continue

| **COLOMBIA** | | | | | | | | |
| --- | --- | --- | --- | --- | --- | --- | --- | --- |
| **Field** | **Count of Authors (#)** | | | **Percentage Distribution (%)** | | | | |
|  | **Q4** | **Q3** | **Q2** | **Q1** | **Q4** | **Q3** | **Q2** | **Q1** |
| AGRI | 1225 | 958 | 113 | 15 | 53.01 | 41.45 | 4.89 | 0.65 |
| ARTS | 129 | 101 | 21 | 7 | 50.00 | 39.15 | 8.14 | 2.71 |
| BIOC | 253 | 275 | 46 | 5 | 43.70 | 47.50 | 7.94 | 0.86 |
| BUSI | 258 | 224 | 48 | 8 | 47.96 | 41.64 | 8.92 | 1.49 |
| CENG | 139 | 127 | 36 | 7 | 44.98 | 41.10 | 11.65 | 2.27 |
| CHEM | 161 | 272 | 68 | 5 | 31.82 | 53.75 | 13.44 | 0.99 |
| COMP | 767 | 612 | 60 | 12 | 52.86 | 42.18 | 4.14 | 0.83 |
| DECI | 8 | 9 | 5 | 1 | 34.78 | 39.13 | 21.74 | 4.35 |
| DENT | 56 | 35 | 6 | 1 | 57.14 | 35.71 | 6.12 | 1.02 |
| EART | 214 | 194 | 41 | 5 | 47.14 | 42.73 | 9.03 | 1.10 |
| ECON | 73 | 100 | 24 | 6 | 35.96 | 49.26 | 11.82 | 2.96 |
| ENER | 168 | 214 | 55 | 8 | 37.75 | 48.09 | 12.36 | 1.80 |
| ENGI | 848 | 717 | 88 | 12 | 50.93 | 43.06 | 5.29 | 0.72 |
| ENVI | 166 | 312 | 55 | 10 | 30.57 | 57.46 | 10.13 | 1.84 |
| HEAL | 12 | 10 | 3 | 0 | 48.00 | 40.00 | 12.00 | 0.00 |
| IMMU | 94 | 91 | 24 | 4 | 44.13 | 42.72 | 11.27 | 1.88 |
| MATE | 159 | 244 | 26 | 0 | 37.06 | 56.88 | 6.06 | 0.00 |
| MATH | 187 | 209 | 26 | 2 | 44.10 | 49.29 | 6.13 | 0.47 |
| MEDI | 3732 | 1341 | 132 | 10 | 71.56 | 25.71 | 2.53 | 0.19 |
| NEUR | 16 | 32 | 16 | 5 | 23.19 | 46.38 | 23.19 | 7.25 |
| NURS | 26 | 42 | 9 | 0 | 33.77 | 54.55 | 11.69 | 0.00 |
| PHAR | 126 | 92 | 17 | 2 | 53.16 | 38.82 | 7.17 | 0.84 |
| PHYS | 596 | 420 | 44 | 5 | 55.96 | 39.44 | 4.13 | 0.47 |
| PSYC | 90 | 100 | 13 | 3 | 43.69 | 48.54 | 6.31 | 1.46 |
| SOCI | 415 | 350 | 55 | 15 | 49.70 | 41.92 | 6.59 | 1.80 |
| VETE | 108 | 53 | 13 | 0 | 62.07 | 30.46 | 7.47 | 0.00 |
| AVERAGE | 385.6 | 274.4 | 40.2 | 5.7 |  |  |  |  |

**Table S5.** continue

| **CHILE** | | | | | | | | |
| --- | --- | --- | --- | --- | --- | --- | --- | --- |
| **Field** | **Count of Authors (#)** | | | **Percentage Distribution (%)** | | | | |
|  | **Q4** | **Q3** | **Q2** | **Q1** | **Q4** | **Q3** | **Q2** | **Q1** |
| AGRI | 1297 | 1512 | 226 | 11 | 42.58 | 49.64 | 7.42 | 0.36 |
| ARTS | 373 | 303 | 50 | 10 | 50.68 | 41.17 | 6.79 | 1.36 |
| BIOC | 542 | 1065 | 153 | 7 | 30.67 | 60.27 | 8.66 | 0.40 |
| BUSI | 49 | 80 | 31 | 9 | 28.99 | 47.34 | 18.34 | 5.33 |
| CENG | 37 | 68 | 27 | 4 | 27.21 | 50.00 | 19.85 | 2.94 |
| CHEM | 289 | 531 | 116 | 6 | 30.68 | 56.37 | 12.31 | 0.64 |
| COMP | 391 | 488 | 75 | 9 | 40.60 | 50.67 | 7.79 | 0.93 |
| DECI | 4 | 12 | 8 | 2 | 15.38 | 46.15 | 30.77 | 7.69 |
| DENT | 75 | 79 | 21 | 1 | 42.61 | 44.89 | 11.93 | 0.57 |
| EART | 363 | 484 | 132 | 12 | 36.63 | 48.84 | 13.32 | 1.21 |
| ECON | 72 | 107 | 56 | 10 | 29.39 | 43.67 | 22.86 | 4.08 |
| ENER | 57 | 74 | 48 | 7 | 30.65 | 39.78 | 25.81 | 3.76 |
| ENGI | 439 | 487 | 98 | 13 | 42.33 | 46.96 | 9.45 | 1.25 |
| ENVI | 254 | 434 | 106 | 8 | 31.67 | 54.11 | 13.22 | 1.00 |
| HEAL | 15 | 27 | 11 | 1 | 27.78 | 50.00 | 20.37 | 1.85 |
| IMMU | 111 | 188 | 31 | 1 | 33.53 | 56.80 | 9.37 | 0.30 |
| MATE | 185 | 324 | 51 | 5 | 32.74 | 57.35 | 9.03 | 0.88 |
| MATH | 162 | 377 | 96 | 5 | 25.31 | 58.91 | 15.00 | 0.78 |
| MEDI | 4337 | 1659 | 165 | 11 | 70.27 | 26.88 | 2.67 | 0.18 |
| NEUR | 69 | 118 | 49 | 9 | 28.16 | 48.16 | 20.00 | 3.67 |
| NURS | 66 | 86 | 18 | 1 | 38.60 | 50.29 | 10.53 | 0.58 |
| PHAR | 87 | 87 | 17 | 4 | 44.62 | 44.62 | 8.72 | 2.05 |
| PHYS | 505 | 885 | 240 | 8 | 30.83 | 54.03 | 14.65 | 0.49 |
| PSYC | 105 | 174 | 50 | 8 | 31.16 | 51.63 | 14.84 | 2.37 |
| SOCI | 706 | 866 | 173 | 24 | 39.91 | 48.95 | 9.78 | 1.36 |
| VETE | 96 | 86 | 13 | 0 | 49.23 | 44.10 | 6.67 | 0.00 |
| AVERAGE | 411.0 | 407.7 | 79.3 | 7.2 |  |  |  |  |

**Table S5.** continue

| **ECUADOR** | | | | | | | | |
| --- | --- | --- | --- | --- | --- | --- | --- | --- |
| **Field** | **Count of Authors (#)*** | | | **Percentage Distribution (%)** | | | | |
|  | **Q4** | **Q3** | **Q2** | **Q1** | **Q4** | **Q3** | **Q2** | **Q1** |
| AGRI | 373 (84) | 422 (211) | 95 (79) | 3 (3) | 41.8 | 47.3 | 10.6 | 0.3 |
| ARTS | 22 (9) | 11 (7) | 0 (0) | 0 (0) | 66.7 | 33.3 | 0.0 | 0.0 |
| BIOC | 63 (12) | 85 (44) | 15 (14) | 2 (2) | 38.2 | 51.5 | 9.1 | 1.2 |
| BUSI | 90 (16) | 34 (15) | 5 (2) | 0 (0) | 69.8 | 26.4 | 3.9 | 0.0 |
| CENG | 14 (5) | 9 (4) | 2 (2) | 0 (0) | 56.0 | 36.0 | 8.0 | 0.0 |
| CHEM | 20 (1) | 54 (20) | 15 (14) | 2 (2) | 22.0 | 59.3 | 16.5 | 2.2 |
| COMP | 202 (94) | 276 (122) | 35 (28) | 2 (2) | 39.2 | 53.6 | 6.8 | 0.4 |
| DECI | 5 (1) | 2 (0) | 1 (1) | 0 (0) | 62.5 | 25.0 | 12.5 | 0.0 |
| DENT | 3 (0) | 4 (3) | 1 (1) | 1 (1) | 33.3 | 44.4 | 11.1 | 11.1 |
| EART | 80 (15) | 84 (41) | 34 (26) | 2 (2) | 40.0 | 42.0 | 17.0 | 1.0 |
| ECON | 6 (0) | 12 (4) | 6 (6) | 4 (4) | 21.4 | 42.9 | 21.4 | 14.3 |
| ENER | 45 (12) | 40 (14) | 12 (4) | 0 (0) | 46.4 | 41.2 | 12.4 | 0.0 |
| ENGI | 230 (44) | 130 (46) | 31 (24) | 0 (0) | 58.8 | 33.2 | 7.9 | 0.0 |
| ENVI | 103 (29) | 160 (76) | 42 (33) | 2 (2) | 33.6 | 52.1 | 13.7 | 0.7 |
| HEAL | 6 (2) | 2 (1) | 2 (1) | 0 (0) | 60.0 | 20.0 | 20.0 | 0.0 |
| IMMU | 30 (5) | 30 (19) | 5 (5) | 0 (0) | 46.2 | 46.2 | 7.7 | 0.0 |
| MATE | 25 (4) | 42 (18) | 10 (6) | 1 (1) | 32.1 | 53.8 | 12.8 | 1.3 |
| MATH | 71 (12) | 32 (8) | 9 (7) | 1 (1) | 62.8 | 28.3 | 8.0 | 0.9 |
| MEDI | 685 (192) | 244 (133) | 47 (46) | 6 (5) | 69.8 | 24.8 | 4.8 | 0.6 |
| NEUR | 29 (4) | 3 (1) | 3 (2) | 0 (0) | 82.9 | 8.6 | 8.6 | 0.0 |
| NURS | 6 (2) | 4 (1) | 0 (0) | 0 (0) | 60.0 | 40.0 | 0.0 | 0.0 |
| PHAR | 70 (18) | 34 (14) | 5 (3) | 0 (0) | 64.2 | 31.2 | 4.6 | 0.0 |
| PHYS | 45 (10) | 147 (98) | 26 (24) | 1 (1) | 20.5 | 67.1 | 11.9 | 0.5 |
| PSYC | 12 (1) | 6 (3) | 4 (3) | 0 (0) | 54.5 | 27.3 | 18.2 | 0.0 |
| SOCI | 178 (30) | 105 (37) | 31 (24) | 8 (8) | 55.3 | 32.6 | 9.6 | 2.5 |
| VETE | 47 (12) | 14 (4) | 3 (3) | 0 (0) | 73.4 | 21.9 | 4.7 | 0.0 |
| AVERAGE | 94.6 (23.6) | 76.4 (36.3) | 17.0 (13.8) | 1.3 (1.3) |  |  |  |  |

* Data in parentheses are the A1-A2 distribution difference in each range quarter.

**Table S5.** continue

| **PARAGUAY** | | | | | | | | |
| --- | --- | --- | --- | --- | --- | --- | --- | --- |
| **Field** | **Count of Authors (#)** | | | **Percentage Distribution (%)** | | | | |
|  | **Q4** | **Q3** | **Q2** | **Q1** | **Q4** | **Q3** | **Q2** | **Q1** |
| AGRI | 40 | 30 | 1 | 0 | 56.34 | 42.25 | 1.41 | 0.00 |
| ARTS | 1 | 0 | 0 | 0 | 100.00 | 0.00 | 0.00 | 0.00 |
| BIOC | 6 | 9 | 1 | 0 | 37.50 | 56.25 | 6.25 | 0.00 |
| BUSI | 5 | 0 | 0 | 0 | 100.00 | 0.00 | 0.00 | 0.00 |
| CENG | 0 | 0 | 0 | 1 | 0.00 | 0.00 | 0.00 | 100.00 |
| CHEM | 2 | 2 | 0 | 0 | 50.00 | 50.00 | 0.00 | 0.00 |
| COMP | 27 | 21 | 2 | 1 | 52.94 | 41.18 | 3.92 | 1.96 |
| DECI | 0 | 0 | 0 | 0 | 0.00 | 0.00 | 0.00 | 0.00 |
| DENT | 3 | 3 | 3 | 0 | 33.33 | 33.33 | 33.33 | 0.00 |
| EART | 4 | 1 | 0 | 0 | 80.00 | 20.00 | 0.00 | 0.00 |
| ECON | 0 | 1 | 0 | 0 | 0.00 | 100.00 | 0.00 | 0.00 |
| ENER | 2 | 1 | 2 | 1 | 33.33 | 16.67 | 33.33 | 16.67 |
| ENGI | 12 | 9 | 4 | 0 | 48.00 | 36.00 | 16.00 | 0.00 |
| ENVI | 7 | 4 | 0 | 0 | 63.64 | 36.36 | 0.00 | 0.00 |
| HEAL | 0 | 0 | 0 | 0 | 0.00 | 0.00 | 0.00 | 0.00 |
| IMMU | 13 | 1 | 1 | 0 | 86.67 | 6.67 | 6.67 | 0.00 |
| MATE | 0 | 0 | 0 | 0 | 0.00 | 0.00 | 0.00 | 0.00 |
| MATH | 1 | 1 | 1 | 1 | 25.00 | 25.00 | 25.00 | 25.00 |
| MEDI | 154 | 43 | 10 | 1 | 74.04 | 20.67 | 4.81 | 0.48 |
| NEUR | 0 | 1 | 0 | 0 | 0.00 | 100.00 | 0.00 | 0.00 |
| NURS | 1 | 0 | 0 | 0 | 100.00 | 0.00 | 0.00 | 0.00 |
| PHAR | 13 | 12 | 3 | 0 | 46.43 | 42.86 | 10.71 | 0.00 |
| PHYS | 6 | 2 | 4 | 0 | 50.00 | 16.67 | 33.33 | 0.00 |
| PSYC | 3 | 2 | 0 | 0 | 60.00 | 40.00 | 0.00 | 0.00 |
| SOCI | 2 | 2 | 0 | 0 | 50.00 | 50.00 | 0.00 | 0.00 |
| VETE | 2 | 1 | 0 | 0 | 66.67 | 33.33 | 0.00 | 0.00 |
| AVERAGE | 11.7 | 5.6 | 1.2 | 0.2 |  |  |  |  |

**Table S5.** continue

| **PERU** | | | | | | | | |
| --- | --- | --- | --- | --- | --- | --- | --- | --- |
| **Field** | **Count of Authors (#)** | | | **Percentage Distribution (%)** | | | | |
|  | **Q4** | **Q3** | **Q2** | **Q1** | **Q4** | **Q3** | **Q2** | **Q1** |
| AGRI | AGRI | 422 | 277 | 41 | 3 | 56.80 | 37.28 | 5.52 |
| ARTS | ARTS | 22 | 27 | 4 | 1 | 40.74 | 50.00 | 7.41 |
| BIOC | BIOC | 61 | 74 | 14 | 1 | 40.67 | 49.33 | 9.33 |
| BUSI | BUSI | 31 | 35 | 13 | 6 | 36.47 | 41.18 | 15.29 |
| CENG | CENG | 4 | 3 | 1 | 0 | 50.00 | 37.50 | 12.50 |
| CHEM | CHEM | 26 | 22 | 2 | 0 | 52.00 | 44.00 | 4.00 |
| COMP | COMP | 296 | 117 | 12 | 1 | 69.48 | 27.46 | 2.82 |
| DECI | DECI | 1 | 2 | 0 | 1 | 25.00 | 50.00 | 0.00 |
| DENT | DENT | 49 | 29 | 7 | 0 | 57.65 | 34.12 | 8.24 |
| EART | EART | 72 | 74 | 15 | 1 | 44.44 | 45.68 | 9.26 |
| ECON | ECON | 9 | 15 | 13 | 3 | 22.50 | 37.50 | 32.50 |
| ENER | ENER | 33 | 11 | 1 | 1 | 71.74 | 23.91 | 2.17 |
| ENGI | ENGI | 206 | 113 | 9 | 6 | 61.68 | 33.83 | 2.69 |
| ENVI | ENVI | 79 | 69 | 18 | 3 | 46.75 | 40.83 | 10.65 |
| HEAL | HEAL | 3 | 2 | 0 | 0 | 60.00 | 40.00 | 0.00 |
| IMMU | IMMU | 48 | 26 | 4 | 0 | 61.54 | 33.33 | 5.13 |
| MATE | MATE | 29 | 27 | 4 | 1 | 47.54 | 44.26 | 6.56 |
| MATH | MATH | 22 | 15 | 6 | 1 | 50.00 | 34.09 | 13.64 |
| MEDI | MEDI | 1365 | 469 | 45 | 6 | 72.41 | 24.88 | 2.39 |
| NEUR | NEUR | 3 | 2 | 0 | 0 | 60.00 | 40.00 | 0.00 |
| NURS | NURS | 4 | 4 | 1 | 0 | 44.44 | 44.44 | 11.11 |
| PHAR | PHAR | 29 | 21 | 4 | 0 | 53.70 | 38.89 | 7.41 |
| PHYS | PHYS | 103 | 54 | 6 | 3 | 62.05 | 32.53 | 3.61 |
| PSYC | PSYC | 21 | 21 | 5 | 1 | 43.75 | 43.75 | 10.42 |
| SOCI | SOCI | 153 | 97 | 21 | 4 | 55.64 | 35.27 | 7.64 |
| VETE | VETE | 171 | 61 | 1 | 0 | 73.39 | 26.18 | 0.43 |
| AVERAGE | 125.5 | 64.1 | 9.5 | 1.7 |  |  |  |  |

**Table S5.** continue

| **URUGUAY** | | | | | | | | |
| --- | --- | --- | --- | --- | --- | --- | --- | --- |
| **Field** | **Count of Authors (#)** | | | **Percentage Distribution (%)** | | | | |
|  | **Q4** | **Q3** | **Q2** | **Q1** | **Q4** | **Q3** | **Q2** | **Q1** |
| AGRI | 287 | 364 | 36 | 2 | 41.65 | 52.83 | 5.22 | 0.29 |
| ARTS | 17 | 15 | 2 | 0 | 50.00 | 44.12 | 5.88 | 0.00 |
| BIOC | 114 | 241 | 22 | 2 | 30.08 | 63.59 | 5.80 | 0.53 |
| BUSI | 4 | 12 | 3 | 1 | 20.00 | 60.00 | 15.00 | 5.00 |
| CENG | 3 | 11 | 1 | 0 | 20.00 | 73.33 | 6.67 | 0.00 |
| CHEM | 24 | 88 | 7 | 2 | 19.83 | 72.73 | 5.79 | 1.65 |
| COMP | 76 | 82 | 7 | 2 | 45.51 | 49.10 | 4.19 | 1.20 |
| DECI | 2 | 0 | 0 | 0 | 100.00 | 0.00 | 0.00 | 0.00 |
| DENT | 3 | 3 | 0 | 0 | 50.00 | 50.00 | 0.00 | 0.00 |
| EART | 23 | 44 | 14 | 1 | 28.05 | 53.66 | 17.07 | 1.22 |
| ECON | 4 | 19 | 5 | 3 | 12.90 | 61.29 | 16.13 | 9.68 |
| ENER | 18 | 22 | 4 | 0 | 40.91 | 50.00 | 9.09 | 0.00 |
| ENGI | 60 | 34 | 2 | 1 | 61.86 | 35.05 | 2.06 | 1.03 |
| ENVI | 25 | 69 | 17 | 2 | 22.12 | 61.06 | 15.04 | 1.77 |
| HEAL | 0 | 0 | 0 | 0 | 0.00 | 0.00 | 0.00 | 0.00 |
| IMMU | 60 | 62 | 12 | 0 | 44.78 | 46.27 | 8.96 | 0.00 |
| MATE | 16 | 22 | 2 | 1 | 39.02 | 53.66 | 4.88 | 2.44 |
| MATH | 29 | 33 | 8 | 2 | 40.28 | 45.83 | 11.11 | 2.78 |
| MEDI | 536 | 225 | 23 | 1 | 68.28 | 28.66 | 2.93 | 0.13 |
| NEUR | 17 | 34 | 11 | 0 | 27.42 | 54.84 | 17.74 | 0.00 |
| NURS | 1 | 1 | 0 | 1 | 33.33 | 33.33 | 0.00 | 33.33 |
| PHAR | 24 | 34 | 5 | 0 | 38.10 | 53.97 | 7.94 | 0.00 |
| PHYS | 33 | 54 | 11 | 0 | 33.67 | 55.10 | 11.22 | 0.00 |
| PSYC | 10 | 12 | 4 | 1 | 37.04 | 44.44 | 14.81 | 3.70 |
| SOCI | 40 | 55 | 12 | 3 | 36.36 | 50.00 | 10.91 | 2.73 |
| VETE | 40 | 53 | 7 | 1 | 39.60 | 52.48 | 6.93 | 0.99 |
| AVERAGE | 56.4 | 61.1 | 8.3 | 1.0 |  |  |  |  |

**Table S5.** continue

| **VENEZUELA** | | | | | | | | |
| --- | --- | --- | --- | --- | --- | --- | --- | --- |
| **Field** | **Count of Authors (#)** | | | **Percentage Distribution (%)** | | | | |
|  | **Q4** | **Q3** | **Q2** | **Q1** | **Q4** | **Q3** | **Q2** | **Q1** |
| AGRI | 665 | 219 | 17 | 2 | 73.64 | 24.25 | 1.88 | 0.22 |
| ARTS | 37 | 15 | 2 | 3 | 64.91 | 26.32 | 3.51 | 5.26 |
| BIOC | 126 | 54 | 5 | 1 | 67.74 | 29.03 | 2.69 | 0.54 |
| BUSI | 39 | 9 | 3 | 0 | 76.47 | 17.65 | 5.88 | 0.00 |
| CENG | 60 | 30 | 5 | 0 | 63.16 | 31.58 | 5.26 | 0.00 |
| CHEM | 162 | 92 | 14 | 0 | 60.45 | 34.33 | 5.22 | 0.00 |
| COMP | 108 | 67 | 7 | 1 | 59.02 | 36.61 | 3.83 | 0.55 |
| DECI | 1 | 0 | 0 | 0 | 100.00 | 0.00 | 0.00 | 0.00 |
| DENT | 7 | 1 | 1 | 0 | 77.78 | 11.11 | 11.11 | 0.00 |
| EART | 82 | 39 | 7 | 0 | 64.06 | 30.47 | 5.47 | 0.00 |
| ECON | 1 | 9 | 6 | 1 | 5.88 | 52.94 | 35.29 | 5.88 |
| ENER | 43 | 25 | 4 | 0 | 59.72 | 34.72 | 5.56 | 0.00 |
| ENGI | 232 | 67 | 13 | 0 | 74.36 | 21.47 | 4.17 | 0.00 |
| ENVI | 62 | 27 | 6 | 0 | 65.26 | 28.42 | 6.32 | 0.00 |
| HEAL | 0 | 0 | 0 | 1 | 0.00 | 0.00 | 0.00 | 100.00 |
| IMMU | 75 | 38 | 1 | 0 | 65.79 | 33.33 | 0.88 | 0.00 |
| MATE | 140 | 68 | 7 | 2 | 64.52 | 31.34 | 3.23 | 0.92 |
| MATH | 78 | 54 | 8 | 0 | 55.71 | 38.57 | 5.71 | 0.00 |
| MEDI | 1375 | 247 | 22 | 5 | 83.38 | 14.98 | 1.33 | 0.30 |
| NEUR | 17 | 9 | 2 | 1 | 58.62 | 31.03 | 6.90 | 3.45 |
| NURS | 19 | 12 | 3 | 0 | 55.88 | 35.29 | 8.82 | 0.00 |
| PHAR | 54 | 45 | 0 | 0 | 54.55 | 45.45 | 0.00 | 0.00 |
| PHYS | 132 | 89 | 9 | 1 | 57.14 | 38.53 | 3.90 | 0.43 |
| PSYC | 11 | 6 | 0 | 0 | 64.71 | 35.29 | 0.00 | 0.00 |
| SOCI | 134 | 54 | 10 | 6 | 65.69 | 26.47 | 4.90 | 2.94 |
| VETE | 153 | 22 | 0 | 0 | 87.43 | 12.57 | 0.00 | 0.00 |
| AVERAGE | 146.7 | 49.9 | 5.8 | 0.9 |  |  |  |  |
